# Diagnostic performance of a Rapid Tick exposure Test (RaTexT^®^) to detect acaricide resistance in cattle ticks in East Africa

**DOI:** 10.1186/s13071-025-06995-6

**Published:** 2025-08-11

**Authors:** Frans Jongejan, Yakob Nagagi, Violet Temba, Dennis Muhanguzi, Patrick Vudriko, Joseph Byaruhanga, Maria Tumwebaze, Frank Mwiine, Pierre-Marie Borne, Marie Ducrotoy, Marjorie Bouchier, Laura Berger, Laura Homminga, Iris Hulsebos, Alita Petersen, Guilherme Klafke

**Affiliations:** 1 Department of Veterinary Tropical Diseases, Faculty of Veterinary Science, University of Pretoria, Private Bag X04, Onderstepoort, 0110 Republic of South Africa; 2 TBD International BV, BioScience Center, Runderweg 6, 8219 PK, Lelystad, The Netherlands; 3 College of Veterinary Medicine Animal Resources and Biosecurity (COVAB), Makerere University, Kampala, Uganda; 4 Ceva Santé Animale, 10 Avenue de La Ballastière, 33500 Libourne, France; 5 Instituto de Pesquisas Veterinárias Desidério Finamor, Estrada Do Conde, 6000, Eldorado Do Sul, RS 92990-000 Brazil

**Keywords:** Rapid Tick exposure Test, Resistance Intensity Test, Acaricide resistance, Ticks, Cattle, Tanzania, Uganda

## Abstract

**Background:**

The Rapid Tick Exposure Test (RaTexT^®^) is a new method for detecting acaricide resistance in cattle ticks. This test provides rapid pen-side results based on the exposure of partially engorged adult ticks to a specially designed acaricide-impregnated matrix. RaTexT^®^ has been utilized in Brazil, where it identified resistance to deltamethrin in both laboratory colonies and field strains of *Rhipicephalus microplus*. The resistance levels in adult ticks tested in Brazil corresponded with those in larvae when using the resistance intensity test (RIT), a modification of the FAO-recommended larval packet test. In this paper, RaTexT^®^ was validated in East Africa using laboratory colonies of cattle ticks from Tanzania and field-collected ticks from Uganda. The resistance levels in adult ticks measured by RaTexT^®^ were compared with those in larvae using the RIT against synthetic pyrethroids, organophosphates, and formamidines.

**Methods:**

The diagnostic validation involved 15,400 adult cattle ticks distributed across 110 RaTexT^®^ boxes and approximately 99,000 larval cattle ticks in 110 RIT tests conducted in Tanzania (*n* = 45) and Uganda (*n* = 65). In Tanzania, semi-engorged adult ticks and larvae from two laboratory colonies of *R. decoloratus* and one strain of *R. appendiculatus *were tested using RaTexT^®^ and the RIT. In Uganda, semi-engorged adult *R. decoloratus* ticks were collected from cattle and immediately tested with RaTexT^®^ in the field. The larval progeny of fully engorged ticks, collected simultaneously from the same cattle, were tested six weeks later under laboratory conditions. Statistical analysis consisted of a combination of categorical (Z-test, Kappa) and continuous (Bland–Altman, CCC, regression) agreement analyses between RaTexT^®^ and RIT.

**Results:**

The results of deltamethrin tests with laboratory ticks in Tanzania and field ticks in Uganda were highly consistent, exhibiting the same high resistance level in adults and larvae after 24 h of exposure. The cypermethrin/chlorpyrifos/PBO tests demonstrated that laboratory tick strains were fully susceptible when the exposure time in RaTexT^®^ was extended to 72 h. In Uganda, field strains demonstrated high resistance to cypermethrin/chlorpyrifos/PBO in RaTexT^®^ while showing low resistance in RIT. The chlorfenvinphos tests revealed that laboratory strains of *R. decoloratus* were susceptible after 48 h of exposure in RaTexT^®^. Both tests identified a low resistance level in adults and larvae of the laboratory strain of *R. appendiculatus* ticks. Resistance to chlorfenvinphos was confirmed in *R. decoloratus* collected in the field in Uganda, where the resistance level in RaTexT^®^ consistently exceeded that in RIT. Comparisons of both tests with amitraz showed that laboratory *R.decoloratus* ticks were susceptible after an extended exposure of 96 h. In field ticks, RaTexT^®^ detected resistance against amitraz, with the resistance level in RaTexT^®^ consistently exceeding that in RIT. The two-proportion Z-Test (P > 0.01) indicated that no significant differences existed between the percentage mortality in 72 out of 168 comparisons between RaTexT^®^ and RIT (42.9%). Cohen’s Kappa statistical analysis of the entire dataset demonstrated moderate to substantial agreement between RaTexT^®^ and RIT for detecting resistance in cattle ticks between 48 and 72 h of tick exposure. RaTexT^®^ demonstrated adequate repeatability, since variance between test boxes was negligible. Overall statistical analysis revealed that RaTexT^®^ can serve as a reliable proxy for RIT, provided that exposure time and acaricidal mode of action are adequately considered in the test design.

**Conclusions:**

RaTexT^®^ detected resistance to three different acaricidal classes in one-host and multi-host cattle ticks in East Africa. The test can differentiate between resistant and susceptible ticks and potentially become a useful decision-support tool for tick control management.

**Graphical abstract:**

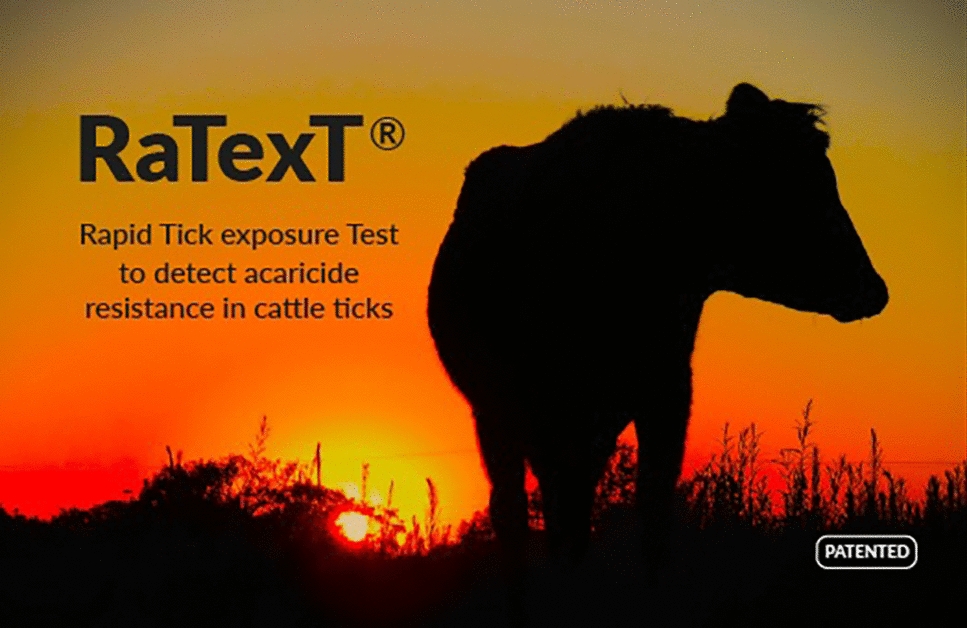

**Supplementary Information:**

The online version contains supplementary material available at 10.1186/s13071-025-06995-6.

## Background

Ticks significantly limit animal production owing to their feeding behavior on cattle, especially in tropical and subtropical regions [1]. Additionally, the diseases transmitted by ticks pose a major obstacle to livestock production. Theileriosis, babesiosis, anaplasmosis, and heartwater are notable cattle health and management problems impacting the livelihoods of farming communities in Africa, Latin America, and Asia [2]. Controlling ticks and tick-borne diseases, particularly in more susceptible and productive European livestock breeds and crossbreeds, relies almost exclusively on acaricides [3–5]. However, many chemical acaricides are toxic and can leave residues in meat and milk, contributing to environmental pollution. The costs associated with their use represent a significant economic burden on the livestock industry. Moreover, frequent exposure of ticks to the same chemicals has led to the development of acaricide resistance [6, 7] in sub-Saharan Africa [8–10], Latin America [11, 12], Asia [13, 14], and Australia [15].

Although acaricide resistance has been reported in a broad range of tick species, one-host *Rhipicephalus* ticks develop resistance faster than other tick species. One-host ticks are potentially exposed to acaricides and selected for resistance in a larger percentage of their life cycle, and there are fewer sylvatic hosts, meaning that the refugia is relatively much smaller than for multi-host ticks [3]. *Rhipicephalus microplus* is the primary one-host tick, which accumulates high tick burdens on cattle. In sub-Saharan Africa, *R. microplus* is also known as the Asian blue tick. It initially arrived in East and Southern Africa with Asian cattle, where it has been shown to displace *Rhipicephalus decoloratus*, the indigenous African blue tick [16]. More recently, *R. microplus* has been introduced into the Ivory Coast with tick-infested cattle from Brazil, leading to widespread dissemination throughout West Africa [17–22].

In East Africa, particularly in Tanzania and Uganda, resistance to cattle ticks has been reported against synthetic pyrethroids, organophosphates, and formamidines [8, 23]. Synthetic pyrethroids induce nerve excitation and rapid mortality in ticks, with mutations in the voltage-gated sodium channel gene associated with resistance [24]. Deltamethrin and cypermethrin are the main pyrethroids used for tick control on livestock, dominating the market in Tanzania (41%) and to a lesser extent in Uganda (20.4%) (Table 1). Organophosphates, particularly chlorfenvinphos, are acetylcholinesterase inhibitors with relatively high toxicity to livestock and humans. Consequently, many organophosphates have been withdrawn from use, resulting in market shares of 1.5% and 2% in Tanzania and Uganda, respectively. However, chlorfenvinphos and chlorpyrifos are often combined with synthetic pyrethroids, representing approximately a third of the acaricidal market in both countries. Finally, the formamidines, amitraz, and cymiazole, are toxic to ticks through their main octopamine receptor site, inducing early detachment, overexcitation, and eventual death. Resistance is believed to manifest through target site modification [25, 26]. The relative market share for amitraz is 23.5% in Tanzania and 32.5% in Uganda (Table 1).

**Table 1:** Acaricides used in this study and their relative market share in Tanzania and Uganda

Acaricide	Number of RaTexT^®^—RIT tests relative market share			
	Tanzania	Uganda	Tanzania*	Uganda**
Deltamethrin (0.25 mg/ml)	13	18	41%***	20.4%***
Cypermethrin/Chlorpyrifos/piperonylbutoxide (0.5625 mg/ml/0.9375 mg/ml)	17	20	34%	37.5%
Chlorfenvinphos (1.0 mg/ml)	10	14	1.5%	2.0%
Amitraz (2.0 mg/ml)	5	13	23.5%	32.5%
Total	**45**	**65**		

^*^Source: Tanzania Veterinary Laboratory Agency, Dar es Salaam, Tanzania

^**^Source: National Drug Authority, Kampala, Uganda: macrocyclic lactones (7.5%) are also registered to control cattle ticks

^***^These percentages include deltamethrin, cypermethrin (= alpha-cypermethrin), flumethrin, and cyhalothrin

Testing for acaricide resistance in cattle ticks is crucial for implementing effective acaricide-resistance management strategies. For this purpose, the larval packet test (LPT) has been recommended by the Food and Agriculture Organization (FAO) since it was published in its Plant Protection Bulletin in 1971 [27], a decade after the original description by Stone and Haydock in 1962 [28]. The LPT protocol has been adopted in the 2004 FAO guidelines for resistance management and integrated parasite control in ruminants. It is widely used as an FAO reference test for detecting resistance in ticks [29]. The FAO guidelines for sustainable tick control and acaricide resistance management in livestock were recently updated [30] together with the larval immersion test (LIT) [31–33], the adult immersion test (AIT), and the larval tarsal test (LTT) [34–36]. The LPT has remained the primary method for identifying acaricide resistance since its introduction over 60 years ago. It has proven useful as a laboratory test for detecting resistance against a background of susceptible laboratory strains. However, the LPT is labour-intensive, requires laboratory facilities with reference susceptible tick strains, and takes at least six weeks to obtain results. Consequently, a recent FAO expert consultation on the sustainable management of acaricide resistance in livestock ticks has recommended developing rapid pen-side diagnostic tests to differentiate between tick resistance and malpractice in chemical tick control [37].

Recently, the Rapid Tick exposure Test (RaTexT^®^) was developed, in which partly engorged adult ticks are exposed to a specially designed acaricide-impregnated matrix that delivers rapid pen-side test results. This novel concept has already been evaluated in Brazil using synthetic pyrethroids, where the test correctly identified both susceptible and deltamethrin-resistant *R. microplus* ticks from reference laboratory colonies and field strains [38]. Furthermore, RaTexT^®^ has also been used to differentiate permethrin-resistant from susceptible laboratory ticks of *Rhipicephalus sanguineus* sensu lato [39]. Moreover, the FAO-recommended LPT was modified to the new resistance intensity test (RIT) [40] by adopting the resistance intensity protocol from the latest WHO guidelines for resistance detection in malaria mosquito vectors [41]. This protocol employs 1×, 5×, and 10 × higher concentrations to reveal low, moderate, and high resistance intensity. This allows us to directly compare resistance detection in adult ticks using RaTexT^®^ with that in tick larvae in the RIT, employing the same acaricidal concentrations in both tests. Each RaTexT^®^ box contained compartments without an impregnated matrix to transport fully engorged ticks from the same farm to the laboratory, where, approximately six weeks later, the RIT was performed to test the larval progeny of the same tick populations.

In this study, the diagnostic performance of this adult assay for detecting acaricide resistance in cattle ticks in East Africa was compared with the larval RIT. Both tests were impregnated with four classes of acaricides and a combination product that reflects the current acaricidal market in Tanzania [23] and Uganda [42].

## Methods

### RaTexT^®^ manufacturing

Each RaTexT box contains six rows of four interconnected compartments in which semi-engorged adult ticks are exposed to an acaricide-impregnated matrix. A seventh row of four interconnected compartments without an impregnated matrix is used to transport fully engorged ticks from the same farm to a laboratory where, approximately six weeks later, the RIT can be performed to test the larval progeny of the ticks. Overall, five medium-sized semi-engorged female ticks, between 5 and 8 mm, are the preferred size of *R. microplus* ticks [38]. Smaller ticks are more vulnerable.

The RaTexT^®^ matrix was impregnated with commercial acaricide formulations at the manufacturer’s recommended concentration, as well as at 5 × and 10 × higher concentrations. The following commercial acaricidal products were used: deltamethrin (Vectocid^®^) and cypermethrin/chlorpyrifos/piperonylbutoxide (Vectoclor Plus pour on^®^) (Céva Animal Health, Libourne, France); chlorfenvinphos (Supaphos^®^) and amitraz (Supatraz^®^) (Sanga Vet. Chem, Ltd, Kampala, Uganda). The concentrations of each acaricide, which correlated between RaTexT^®^ and RIT, are provided in Table 1. The selection of each acaricide class reflected their relative market share in Tanzania and Uganda (Table 1).

The nylon matrix was cut into the required format using a laser-driven ScanNCut (Zijlstra, Groningen, The Netherlands) and then impregnated in a separate zip-bag with a mixture of the acaricides in acetone and olive oil (2:1) for each dilution. The control matrix was first impregnated using a diluent of acetone/olive oil. Each acaricide mixture was thoroughly mixed with the matrix pieces inside a zip bag to ensure a homogeneous coating. Subsequently, the matrix pieces were dried overnight in an aluminum tray within a fume hood. Each matrix piece was secured to the bottom of each compartment of the RaTexT^®^ container with a small drop of RTV-1 Elastosil E4 silicone glue. The inside of the lid remained uncovered.

### RaTexT^®^ user protocol

Partly engorged female ticks were manually removed from cattle and collected in a plastic container. The container was then emptied into a shallow metal tray. The ticks were allowed to move around and were selected based on sex, vitality (actively walking), and preferred size. Excess moisture or dirt was left behind, reducing the need for washing, as ticks must be dry when inserted into RaTexT^**®**^. Using a pair of tweezers, five partly engorged female ticks were inserted into each compartment, filling the control compartment first and finishing with the highest acaricide concentration. Afterward, all lids were firmly closed, and the RaTexT^**®**^ box was placed inside a plastic zip bag with a piece of moist tissue. Each box was left at room temperature and out of direct sunlight for 24 h.

After 24 h, ticks were removed from the compartments, starting with the controls to record the number of live and dead ticks. The number of dead ticks was documented in a Data Capture Form. A tick was considered dead or “knocked down” if it lost its ability to walk. This procedure was facilitated by placing each tick inside a small grey circle on the Data Capture Form. Ticks that could walk out of the circle were recorded as alive, while those that could not move even after being stimulated with breath and gently prodded with blunt forceps were marked as dead.

### Resistance intensity test (RIT) manufacturing

The RIT was treated with the same commercial acaricidal formulations used for RaTexT^**®**^ according to the recently described methods [40]. The same acaricides as used for RaTexT were used in the RIT: these were: deltamethrin, cypermethrin/chlorpyrifos/piperonylbutoxide, chlorfenvinphos and amitraz. The manufacturer’s recommended concentrations were used, along with five times and ten times higher concentrations. The dilutions were prepared in a mixture of laboratory-grade acetone and olive oil (2:1). Subsequently, 0.9 ml was applied to 10 × 7 cm Whatman (no.1) quality filter papers in triplicate (Merck Life Science, Amsterdam). The filter papers were then dried in a fume hood for at least one hour, grouped by concentration, sealed in plastic bags, and stored in the dark at room temperature until used. A different concentration of acaricide, along with control packets, was prepared for each field isolate.

Engorged female ticks were collected from cattle and incubated in 150 mm glass rearing vials sealed with stoppers that allowed ventilation. These vials were placed in an incubator at 28 °C, with relative humidity maintained between 85 and 95%. Larvae aged 2 to 4 weeks were used for the experiments.

### Resistance intensity test (RIT) user protocol

A tick-proof working station was filled with water containing a 1% cleaning and disinfectant solution, and double-sided tape was fitted around the edge as an additional barrier. Using a fine brush, approximately 50 ticks were added to each packet. Control packets were filled first, and those with the highest acaricide concentration were filled last. The sealed larval packets were placed in a container where a saturated atmosphere of potassium sulphate was maintained (approximately 85% relative humidity). Incubation occurred at room temperature for 24 h. After incubation, each packet was opened, and both live and dead larvae were counted, starting with the control and progressing to the highest concentration. After counting, all packets containing dead and alive ticks were disposed of into the water reservoir, followed by adding ethanol, and kept inside the working station with the lid on. The criterion for mortality was the larvae’s inability to walk after stimulation by the operator breathing gently on them after the packets were opened.

### Study design

It has been previously shown that that RaTexT^®^ can detect resistance to synthetic pyrethroids in adult *R. microplus* ticks collected in Brazil by exposing them for 24 h to an acaricide-impregnated matrix [38]. The purpose of this study was to demonstrate the ability of RaTexT^®^ to detect resistance to synthetic pyrethroids, organophosphates, combinations of organophosphates and synthetic pyrethroids, and formamidines in other species of African cattle ticks. The comparison of resistant and susceptible adult ticks from tick colonies, as previously performed in Brazil, was not possible in East Africa due to the lack of susceptible tick colonies in the participating laboratories. Therefore, it was decided to follow a protocol in which the detection of resistance in adult tick populations was compared with that detected in larval tick populations. To do this, the following assumptions and adaptations were made:

- 1. We decided to expose adult and larval ticks to the same acaricidal concentrations to allow for a direct comparison of different life cycle stages, whereby it was not assumed that the level of resistance would necessarily be the same.
- 2. We adopted the resistance intensity protocol from WHO guidelines for mosquitoes, using a recommended concentration (1×), plus a 5 × and 10 × concentrated dose, to be able to determine the intensity of acaricide resistance in livestock ticks by RaTexT^®^.
- 3. As a consequence of points 1 and 2, we modified the larval packet test by introducing the same intensity protocol using 1×, 5 ×, and 10 × higher concentrations, leading to the resistance intensity test (RIT).
- 4. Although the criteria for efficacy do not correspond with criteria for resistance, we used the WAAVP guidelines with 90% field efficacy of acaricides as a starting point in a novel bioassay for which guidelines remain to be developed [43].
- 5. We chose formulated acaricidal compounds rather than technical grade actives, since we were to develop a field-based bioassay wherein the behavior of semi-engorged ticks would be similar to ticks exposed to the formulated products used on cattle.
- 6. We used the 90% cut-off in a novel decision table to determine whether ticks were susceptible or exhibited low, medium, or high resistance level (Table 2).
- 7. A final practical adaptation concerned the replacement of trichloroethylene (TCE) by acetone, since TCE is carcinogenic and has been removed from the market. Acetone combined with olive oil solubilized all active ingredients and was found a suitable alternative (unpublished data).

**Table 2:** A decision table to determine the resistance level of ticks exposed to different concentrations of acaricides in RaTexT® and RIT

1	A	Mortality in control ≤ 10%	Use Abbott’s formula for correction: continue at 2
	B	Mortality in control > 10%	Disregard and start again
2	A	Mortality at 1 × RD ≥ 90%	**Susceptible**
	B	Mortality at 1 × RD < 90%	Continue at 3
3	A	Mortality at 5 × RD ≥ 90%	**Low resistance**
	B	Mortality at 5 × RD < 90%	Continue at 4
4	A	Mortality at 10 × RD ≥ 90%	**Moderate resistance**
	B	Mortality at 10 × RD < 90%	**High resistance**

### Country-specific study design: Tanzania

**Fig. 1:**
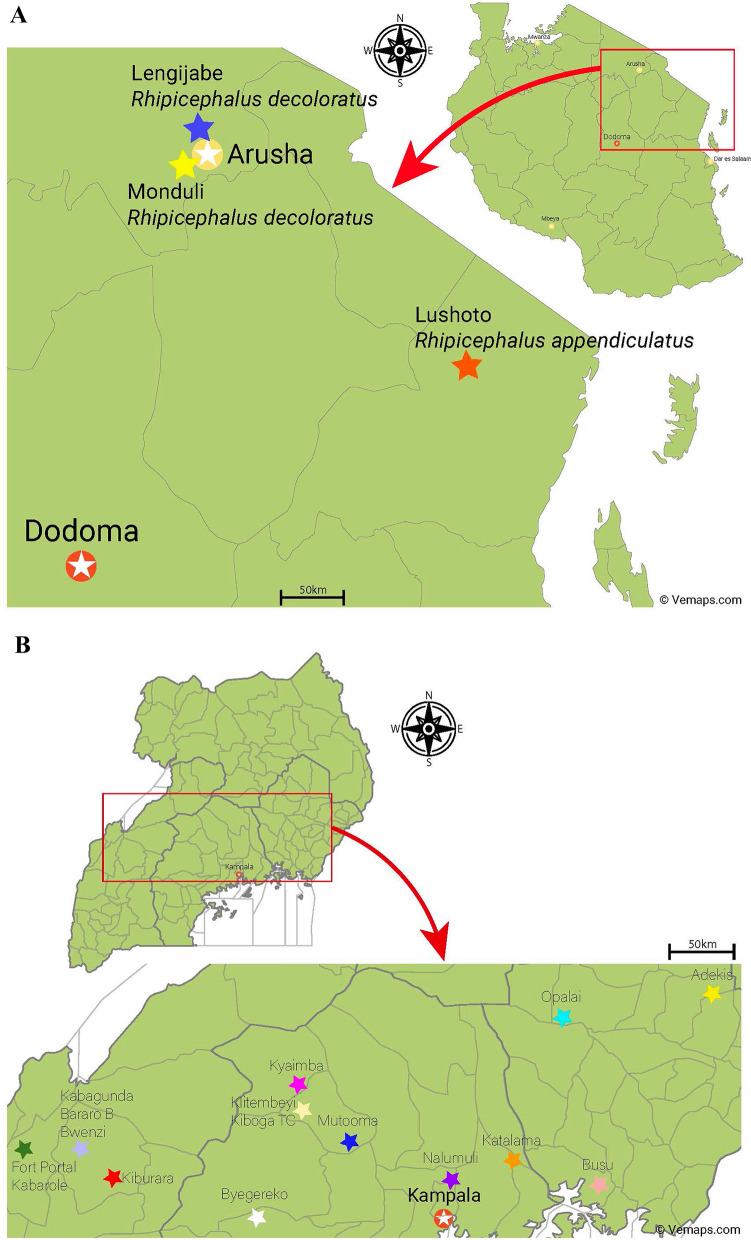
**A** Map of Tanzania with tick collection sites. **B** Map of Uganda with tick collection sites

In Tanzania, the protocol was a strict laboratory method utilizing laboratory-maintained adult and larval ticks. *Rhipicephalus decoloratus* ticks were collected from cattle in 2023 in Monduli district, where tick control practices among pastoral communities were recently described [44] (Fig. 1A). The ticks were initially maintained on rabbits, but from 2024 onwards, they were transitioned to experimental calves to increase the colonies. In the studies, *R. decoloratus* (Monduli strain) was in its 5th and 6th laboratory generations. A second strain of *R. decoloratus* was collected from cattle in Lengijabe in 2021 (Fig. 1A) and was used in its 9th and 10th laboratory generations. A third laboratory strain consisted of *Rhipicephalus appendiculatu*s ticks collected from cattle in Lushoto, Tanga, in 2002 (Fig. 1), which had reached its 23rd laboratory generation when used. Adult and larval ticks derived from all three strains were utilised to compare the mortality of adult ticks measured in RaTexT^®^ with the larval mortality observed in RIT. A total of 45 RaTexT-RIT test comparisons were conducted, divided among the different products listed in Table 1.

### Country-specific study design: Uganda

In Uganda, the protocol was revised to include testing adult ticks in RaTexT® in the field, followed by testing their subsequent larval generation ticks in the laboratory, due to the lack of tick-breeding facilities.

In a previous study, we discovered the presence of *R. microplus* in Serere district of southeastern Uganda, where this invasive tick has replaced the indigenous *R. decoloratus* [45]. *R. microplus* has also been identified in Uganda’s Central, Karamoja, and West Nile regions [46]. The districts visited during this study were located in the Central and Western parts of Uganda, historically reporting acaricide-resistant *R. decoloratus*, without any previous records of *R. microplus* [47] (Fig. 1B). Moreover, no recent records concerning resistance were available at the start of the study, which was expected and highlights the need for a rapid test. Nevertheless, acaricide resistance is more pronounced in the central and southwestern parts of the country, where the local cattle population has been selected for improved production performance traits and is kept under a strict and frequent acaricidal treatment regime[8, 42]. In contrast, indigenous, tick-resistant cattle in the eastern part of the country require fewer acaricides. Therefore, these districts were largely excluded from this validation process (except Adekis and Apalai sites to demonstrate this) because less resistance would be anticipated. Semi-engorged ticks were collected from cattle at different farms and exposed to RaTexT^®^. In addition, fully engorged ticks, which were not used in RaTexT^®^, were collected from cattle at the same farms and allowed to produce a larval progeny. Approximately 6 weeks later, the RIT was applied to the laboratory conditions at Makerere University in Kampala. A total of 65 RaTexT-RIT test comparisons were conducted, divided among the different products listed in Table 1.

### Statistical comparison of RaTexT^®^ and RIT.

In both tests, Abbott’s formula was used to correct for mortality of 10% or less:

$$Corrected mortality percentage= \frac{\% test mortality-\% control mortality}{100-\% control mortality}x 100$$

#### RaTexT^®^-RIT validation

The results were statistically analyzed using a normally distributed dataset with the two-proportion Z-test. For this purpose, the two-proportion Z-test required a minimum sample size of 30 adult ticks per concentration. The following null hypothesis was tested: H_0_: μ_1_ = μ_2_ (where both population proportions are equal). The formula used to calculate the Z statistic was: $Z-value=\frac{{{\varvec{p}}}_{1}-{{\varvec{p}}}_{2}}{\sqrt{{\varvec{P}}(1-{\varvec{P}})(\frac{1}{{{\varvec{n}}}_{1}}+\frac{1}{{{\varvec{n}}}_{2}})}}$ Where p_1_ and p_2_ are the sample proportions, n_1_ and n_2_ are the sample sizes, and p is the total pooled proportion calculated as $P=\frac{p1+p2}{n1+n2}$. The *P*-value was derived from the Z-value using the Z table, and the null hypothesis was rejected if the corresponding *P*-value was less than the significance level (*P* < 0.01).

#### Cohen’s Kappa analysis

This was conducted to assess the level of agreement between RaTexT^®^ and RIT in detecting resistance using the definitions outlined in Table 2. An unweighted Kappa model was executed using VassarStats (http://www.vassarstats.net) to calculate the observed Kappa value. The Kappa values were interpreted as follows: ≤ 0 indicates no agreement, 0.01–0.20 represents none to slight agreement, 0.21–0.40 is considered fair, 0.41–0.60 moderate, 0.61–0.80 substantial, and 0.81–1.00 corresponds to almost perfect agreement.

### Repeatability Assessment

The repeatability of the RaTexT^®^ assay was evaluated using sets of independent tests, with each RaTexT^®^ box considered a distinct experimental unit. Each box contained 24 test chambers, including six replicates for each of the three concentrations (1 × , 5 × , and 10 ×) and appropriate negative controls. For each concentration and time point, the numbers of live and dead ticks across the six corresponding chambers within a given box were pooled to compute a single mortality value. This aggregation allowed for consistent comparison of independent replicates and served as the basis for the repeatability analysis. Only experiments that included at least six independent RaTexT^®^ boxes were considered for statistical analysis to ensure robustness of the variance component estimation. Repeatability was quantified using the intraclass correlation coefficient (ICC), derived from a linear mixed-effects model (LMM) with a random intercept for each independent RaTexT^®^ box (replicate). The LMM was fitted using restricted maximum likelihood (REML) estimation. The ICC was calculated as the ratio between the group variance and the total variance:

$${\text{ICC}}\, = \,\sigma^{{2}} \_{\text{between }}/ \, (\sigma^{{2}} \_{\text{between}}\, + \,\sigma^{{2}} \_{\text{within}})$$

where σ^2^_between is the variance component associated with differences between RaTexT^®^ boxes (random intercept), and σ^2^_within is the residual (within-box) variance. Confidence intervals (95% CI) for ICC estimates were computed using the F-distribution approximation. Analyses were performed in Python (v3.11) using the statsmodels library (v0.14.0), employing the MixedLM class for model fitting and custom routines for ICC and CI computation. Descriptive statistics (mean, standard deviation, and coefficient of variation) were calculated for each concentration using Microsoft Excel.

#### Bland–Altman analysis

To quantitatively assess the agreement between RaTexT^®^ and RIT, we performed the Bland–Altman analysis, simple linear regression, and Lin’s Concordance Correlation Coefficient (CCC) calculations. Mortality rates were recorded at 1 × , 5 × , and 10 × discriminating concentrations, and averaged for each test replicate. The analyses were conducted separately for each exposure duration, allowing for a time-specific evaluation of test performance.

The method was used to calculate the mean difference between RaTexT® and RIT mortalities, along with the 95% limits of agreement (LoA), defined as the mean difference ± 1.96 × standard deviation. Lin’s CCC was used to quantify overall concordance, incorporating both accuracy (closeness to the identity line) and precision (correlation). CCC was calculated as:

$$\rho c \, = {{\left[ {2r \, sx \, sy} \right]} \mathord{\left/ {\vphantom {{\left[ {2r \, sx \, sy} \right]} {\left[ {sx^{2} \, + \,sy^{2} \, + \,\left( {\overline{x} - \overline{y}} \right)} \right]}}} \right. \kern-0pt} {\left[ {sx^{2} \, + \,sy^{2} \, + \,\left( {\overline{x} - \overline{y}} \right)} \right]}}$$

All statistical analyses and visualisations were performed using Python (version 3.11) with the Pandas, NumPy, Matplotlib and scikit-learn libraries. Regression and Bland–Altman plots were generated using matplotlib, with mortality expressed as a percentage corrected for control mortality using Abbott’s formula (see also supplementary data).

## Results

The diagnostic validation of the rapid tick exposure test for detecting acaricide resistance in livestock ticks included 110 RaTexT^®^ boxes and 110 corresponding RIT filter paper tests, which were deployed in Tanzania (*n* = 45) and Uganda (*n* = 65). A total of 15,400 semi-engorged adult cattle ticks were tested in RaTexT^®^ and approximately 99,000 larval cattle ticks in RIT. In Tanzania, adult ticks and their larval offspring, derived from two laboratory colonies of *R. decoloratus* and one strain of *R. appendiculatus,* were tested in RaTexT^®^ and compared with the RIT. In Uganda, semi-engorged adult *R. decoloratus* ticks were collected from cattle and immediately tested by RaTexT^®^ in the field.

 The statistical evaluation of the percentage mortality of laboratory and field ticks in RaTexT^®^ and RIT for deltamethrin, cypermethrin/chlorpyrifos/PBO, chlorfenvinphos, and amitraz, using the two-proportion Z-test along with the conclusions derived from the decision tree, is presented in Tables 3, 4, 5, and 6.

**Fig. 2:**
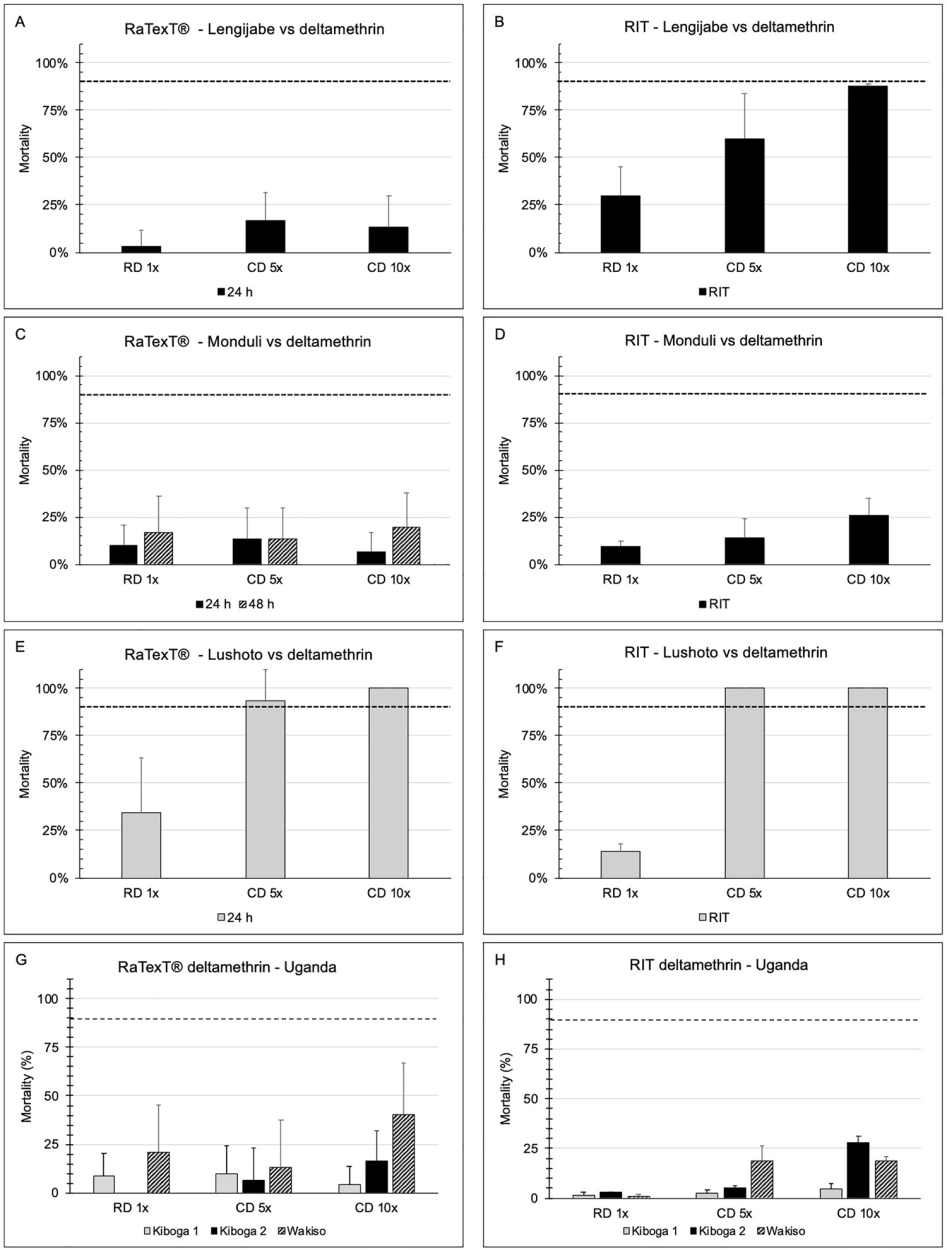

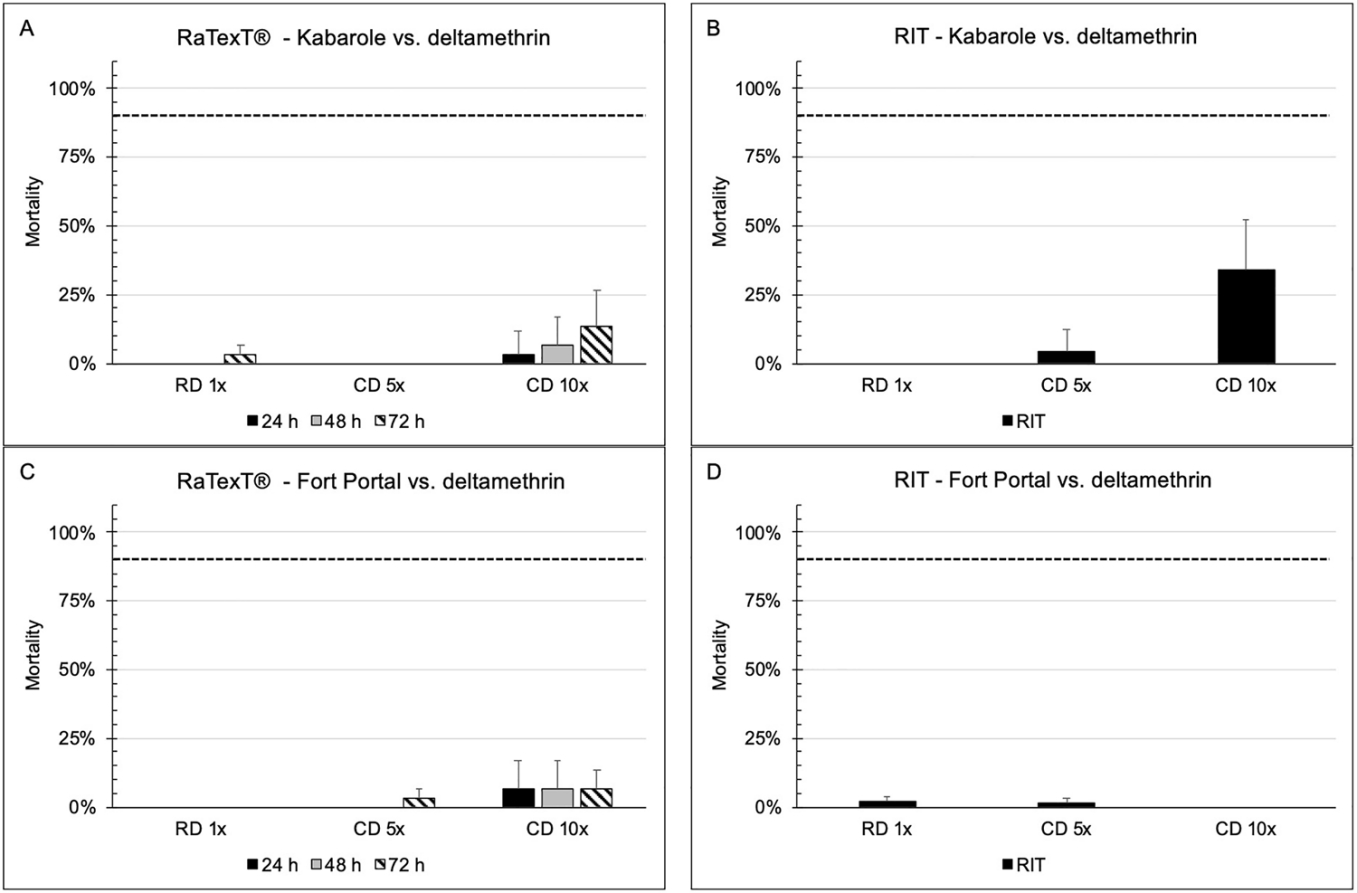
RaTexT-RIT comparison with deltamethrin, against laboratory tick strains from Tanzania (Panels **A** and **B**: *Rhipicephalus decoloratus* Lengijabe strain; **C** and **D**: *R. decoloratus* Monduli strain; **E** and **F**: *Rhipicephalus appendiculatus* Lushoto strain) and *R. decoloratus* field ticks from Uganda (Panels **G** and **H**). cont. RaTexT-RIT comparison with deltamethrin, against field tick strains from Uganda (Panel **A** and **B**: Kabarole; Panel **C** and **D**: Fort Portal)

RaTexT^®^-RIT test results with deltamethrin after 24 h of exposure on laboratory tick strains from Tanzania (Panels A and B: *R. decoloratus* Lengijabe strain; C and D: *R. decoloratus* Monduli strain; E and F: *R. appendiculatus* Lushoto strain) and *R. decoloratus* field ticks from Uganda (Panels G and H) were highly consistent and indicated a high level of resistance using the 90% cut-off value (Fig. 2) (Table 3). A statistical comparison of the percentage mortality in RaTexT^®^ versus RIT, using the two-proportion Z-Test with *P*-values > 0.01, revealed no significant differences in 6 out of 39 test comparisons (Table 3). In certain instances, the exposure time to deltamethrin was extended to 48 and 72 h, which did not result in increased mortality (Fig. 2).

**Table 3:** Statistical comparison of the percentage mortality of ticks from Tanzania and Uganda in RaTexT^®^ and RIT for deltamethrin using the two-proportion Z-test combined with the conclusions derived from the decision tree

Tick isolate	Z-test (*P*-value)									Conclusion (Resistance level)			
	24 h			48 h			72 h						
	RD 1×	CD 5×	CD 10×	RD 1×	CD 5×	CD 10×	RD 1×	CD 5×	CD 10×	RaTexT^®^ 24 h	RaTexT^®^ 48 h	RaTexT^®^ 72 h	RIT
*R. decoloratus* Lengijabe	< 0.001	< 0.001	< 0.001	–	–	–	–	–	–	High	–	–	High
*R. decoloratus* Monduli	0.619*	0.865*	< 0.001	< 0.001	0.865*	0.264*	–	–	–	High	High	–	High
*R. appendiculatus* Lushoto	< 0.001	0.728*	1*	–	–	–	–	–	–	Low	–	–	Low
*R. decoloratus* Kiboga 1	0	0	0	–	–	–	–	–	–	High	–	–	High
*R. decoloratus* Wakiso	< 0.001	0	0	–	–	–	–	–	–	High	–	–	High
*R. decoloratus* Kiboga 2	0	0	0	–	–	–	–	–	–	High	–	–	High
*R. decoloratus* Kabarole	0	< 0.001	< 0.001	0	< 0.001	< 0.001	0	< 0.001	< 0.001	High	High	High	High
*R. decoloratus* Fort Portal	< 0.001	< 0.001	0	< 0.001	< 0.001	0	< 0.001	< 0.001	0	High	High	High	High

Asterisks indicate *P*-value > 0.01 and no significant differences between tests (RaTexT and RIT)

**Fig. 3:**
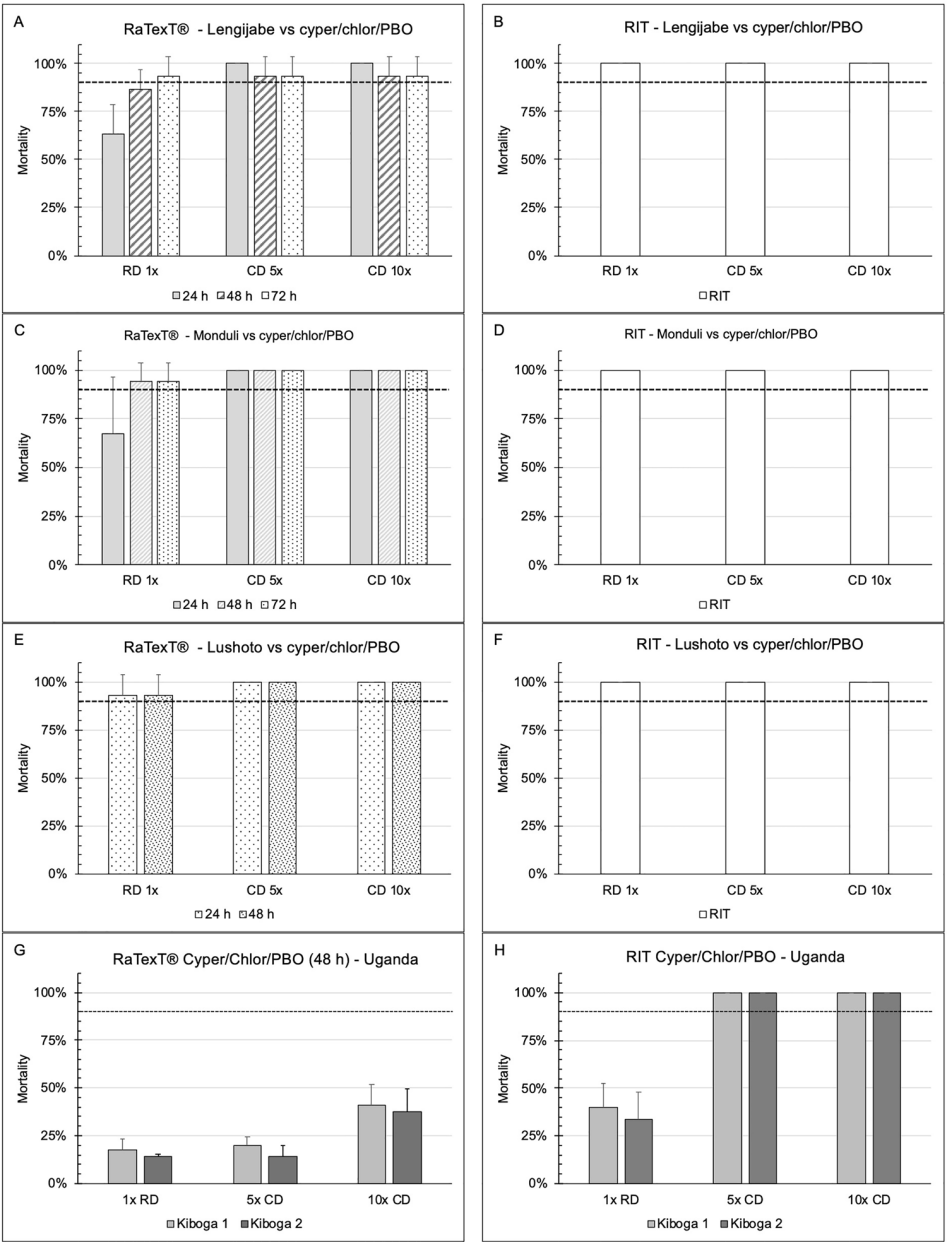
RaTexT-RIT comparison with cypermethrin/chlorpyrifos/PBO, against laboratory tick strains from Tanzania (Panels **A** and **B**: *R. decoloratus* Lengijabe strain; **C** and **D**: *R. decoloratus* Monduli strain; **E** and **F**: *R. appendiculatus* Lushoto strain) and *R. decoloratus* field ticks from Uganda (Panels **G** and **H**)

RaTexT^®^-RIT test results using cypermethrin/chlorpyrifos/PBO against laboratory tick strains from Tanzania (Panels A and B: *R. decoloratus* Lengijabe strain; C and D: *R. decoloratus* Monduli strain; E and F: *R. appendiculatus* Lushoto strain) and *R. decoloratus* field ticks from Uganda (Panels G and H) (Fig. 3) demonstrated that laboratory tick strains were fully susceptible when the exposure time in RaTexT^®^ was extended to 72 h (Fig. 3). In contrast, field strains were highly resistant to cypermethrin/chlorpyrifos/PBO in RaTexT^**®**^ and exhibited low resistance in RIT (Table 4). The two-proportion Z-Test with *P*-values > 0.01 indicated no significant differences between mortality percentages in 26 out of 30 test comparisons (Table 4).

**Table 4:** Statistical comparison of the percentage mortality of ticks from Tanzania and Uganda in RaTexT® and RIT for cypermethrin/chlorpyrifos/PBO using the two-proportion Z-test combined with the conclusions derived from the decision tree

Tick isolate	Z-test (*P*-value)									Conclusion (resistance level)			
	24 h			48 h			72 h						
	RD 1×	CD 5×	CD 10×	RD 1×	CD 5×	CD 10×	RD 1×	CD 5×	CD 10×	RaTexT^®^ 24 h	RaTexT^®^ 48 h	RaTexT^®^ 72 h	RIT
*R. decoloratus* Lengijabe	0.051*	1*	1*	0.736*	1*	1*	1*	1*	1*	Low	Low	Susceptible	Susceptible
*R. decoloratus* Monduli	0.081*	1*	1*	0.737*	1*	1*	0.737*	1*	1*	Low	Susceptible	Susceptible	Susceptible
*R. appendiculatus* Lushoto	**0.728**	1*	1*	**0.728**	1*	1*	–	–	–	Susceptible	Susceptible	–	Susceptible
*R. decoloratus* Kiboga 1	–	–	–	0.739*	< 0.001	0.009	–	–	–	–	High	–	Low
*R. decoloratus* Kiboga 2	–	–	–	0.423*	< 0.001	0.001	–	–	–	–	High	–	Low

Asterisks indicate *P*-value > 0.01 and no significant differences between tests (RaTexT and RIT)

**Fig. 4:**
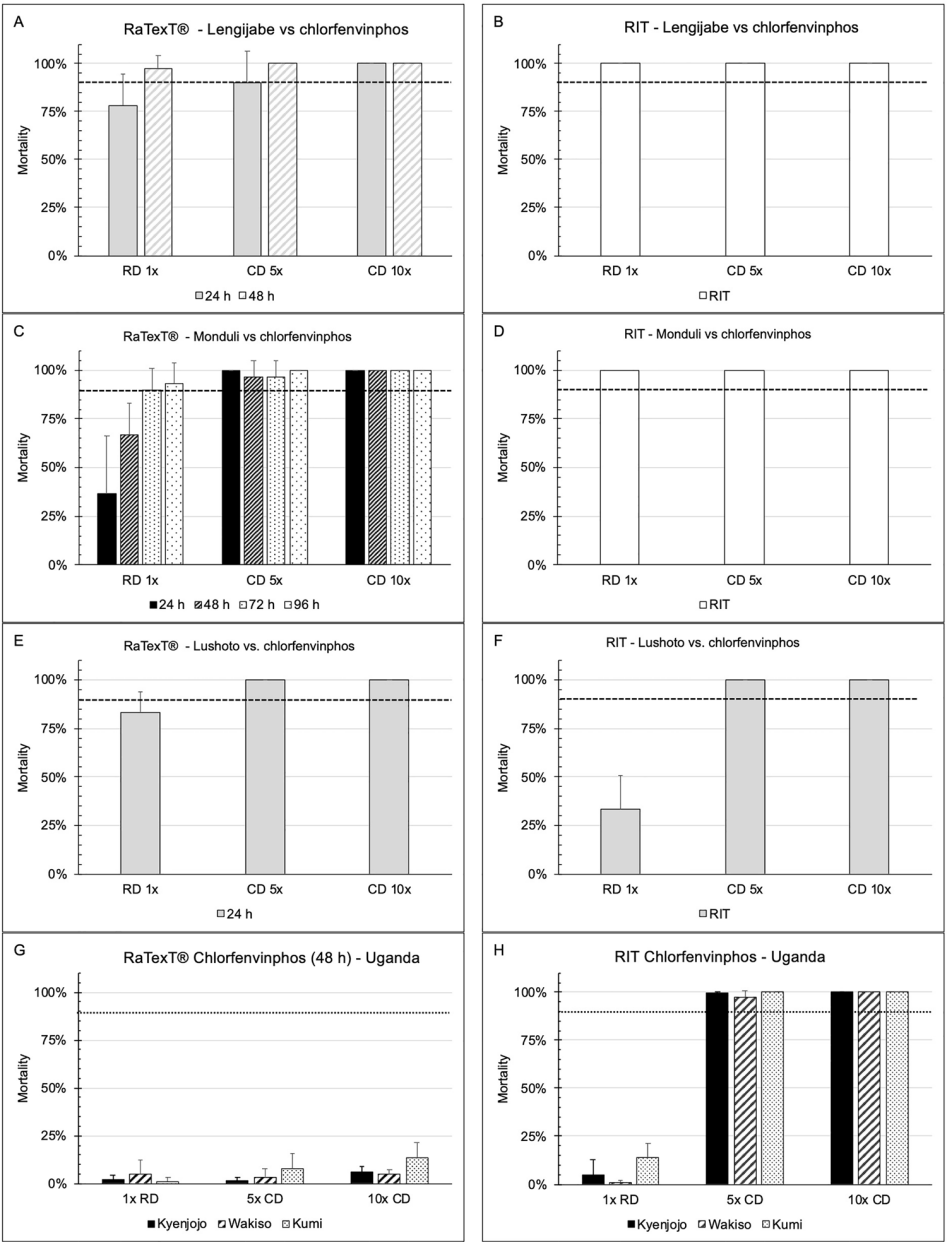

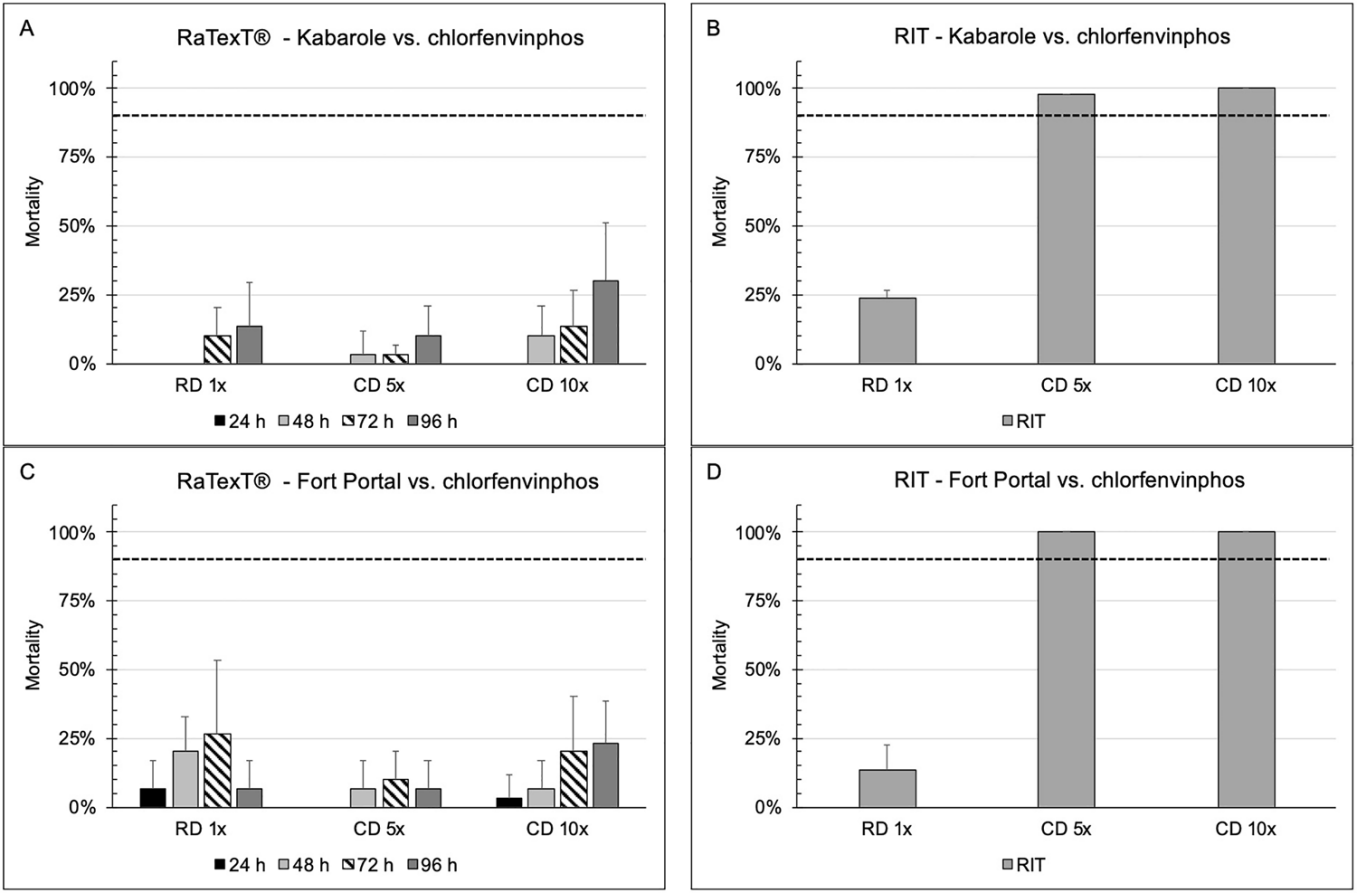
RaTexT-RIT comparison with chlorfenvinphos, against laboratory tick strains from Tanzania (Panels **A** and **B**: *R. decoloratus* Lengijabe strain; **C** and **D**: *R. decoloratus* Monduli strain; **E** and **F**: *R. appendiculatus* Lushoto strain) and *R. decoloratus* field ticks from Uganda (Panels **G** and **H**). cont. RaTexT-RIT comparison with chlorpyriphos, against field tick strains from Uganda (Panel A and B: Kabarole; Panel **C** and **D**: Fort Portal)

The RaTexT^®^-RIT comparison with chlorfenvinphos against laboratory tick strains from Tanzania (Fig. 4: panels A and B: *R. decoloratus* Lengijabe strain; C and D: *R. decoloratus* Monduli strain) revealed that laboratory strains of *R. decoloratus* were susceptible to chlorfenvinphos after an exposure time of 48 h in RaTexT^®^. Both tests indicated a low level of resistance detected in adults and larvae of the laboratory strain of *R. appendiculatus* ticks (Fig. 4, panels E and F). Resistance to chlorfenvinphos was confirmed in *R. decoloratus* collected in the field in Uganda (Fig. 4: panels G and H), where the resistance level in RaTexT® was consistently higher than in RIT (Table 5). The two-proportion Z-Test with *P*-values > 0.01 indicated no significant differences between mortality percentages in 24 out of 54 test comparisons (Table 5).

**Table 5:** Statistical comparison of the percentage mortality of ticks from Tanzania and Uganda in RaTexT® and RIT for chlorfenvinphos using the two-proportion Z-test combined with the conclusions derived from the decision tree

Tick isolate	Z-test (*P*-value)												Conclusion (resistance level)				
	24 h			48 h			72 h			96 h							
	RD 1×	CD 5×	CD 10×	RD 1×	CD 5×	CD 10×	RD 1×	CD 5×	CD 10×	RD 1×	CD 5×	CD 10×	RaTexT^®^ 24 h	RaTexT^®^ 48 h	RaTexT^®^ 72 h	RaTexT^®^ 96 h	RIT
*R. decoloratus* Lengijabe	0.224*	0.602*	1*	0.865*	1*	1*	–	–	–	–	–	–	Low	Susceptible	–	–	Susceptible
*R. decoloratus* Monduli	< 0.001	1*	1*	0.075*	0.863*	1*	0.604*	0.863*	1*	0.731*	1*	1*	Low	Low	Susceptible	Susceptible	Susceptible
*R. appendiculatus* Lushoto	< 0.001	1*	1*	–	–	–	–	–	–	–	–	–	Low	–	–	–	Low
*R. decoloratus* Kyenjojo	–	–	–	0.04*	< 0.001	< 0.001	–	–	–	–	–	–	–	High	–	–	Low
*R. decoloratus* Wakiso	–	–	–	< 0.001	< 0.001	< 0.001	–	–	–	–	–	–	–	High	–	–	Low
*R. decoloratus* Kumi	–	–	–	< 0.001	< 0.001	< 0.001	–	–	–	–	–	–	–	High	–	–	Low
*R. decoloratus* Kabarole	< 0.001	< 0.001	< 0.001	< 0.001	< 0.001	< 0.001	0.035*	< 0.001	< 0.001	0.323*	< 0.001	< 0.001	High	High	High	High	Low
*R. decoloratus* Fort Portal	0.018*	< 0.001	< 0.001	0.001	< 0.001	< 0.001	< 0.001	< 0.001	< 0.001	0.018*	< 0.001	< 0.001	High	High	High	High	Low

Asterisks indicate *P*-value > 0.01 and no significant differences between tests (RaTexT and RIT)

**Fig. 5:**
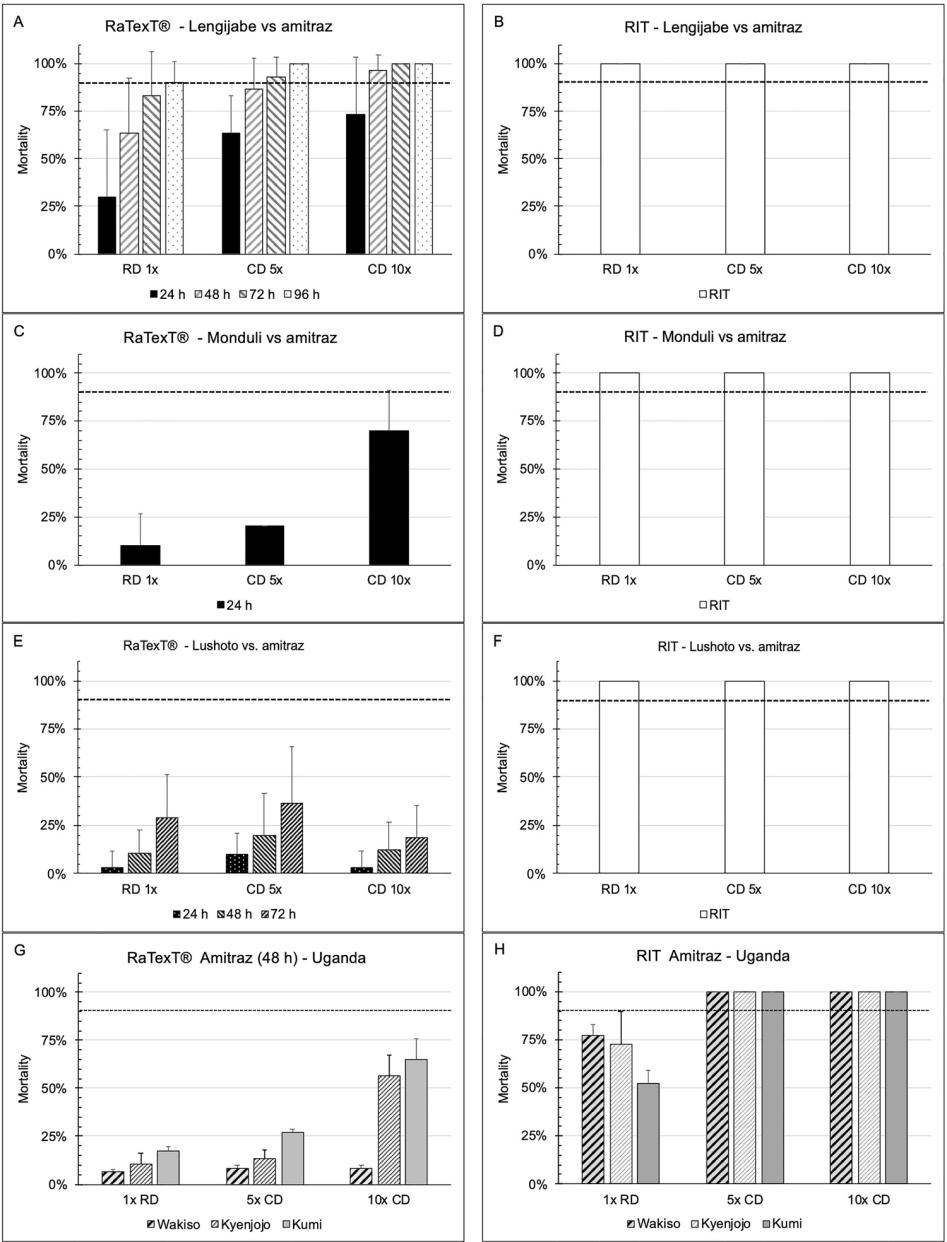

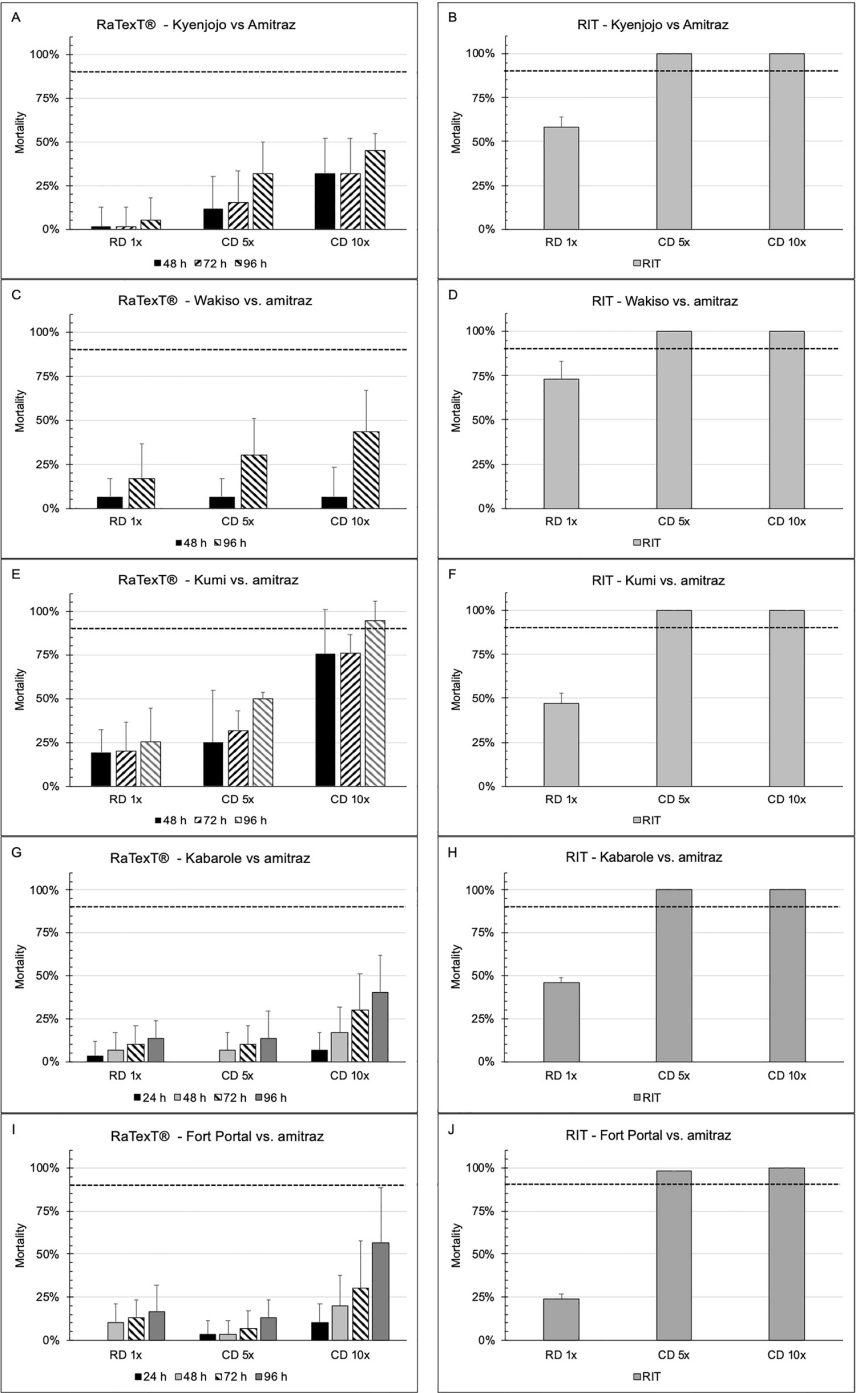
RaTexT-RIT comparison with amitraz, against laboratory tick strains from Tanzania (Panels **A** and **B**: *R. decoloratus* Lengijabe strain; **C** and **D**: *R. decoloratus* Monduli strain; **E** and **F**: *R. appendiculatus* Lushoto strain) and *R. decoloratus* field ticks from Uganda (panels G and H). cont. RaTexT-RIT comparison with amitraz against field tick strains from Uganda (panels A and B: Kyenjojo; panels **C** and **D**: Wakiso; panels **E** and **F**: Kumi; panels **G** and **H**: Kabarole; panels **I** and **J**: Fort Portal)

**Fig. 6:**
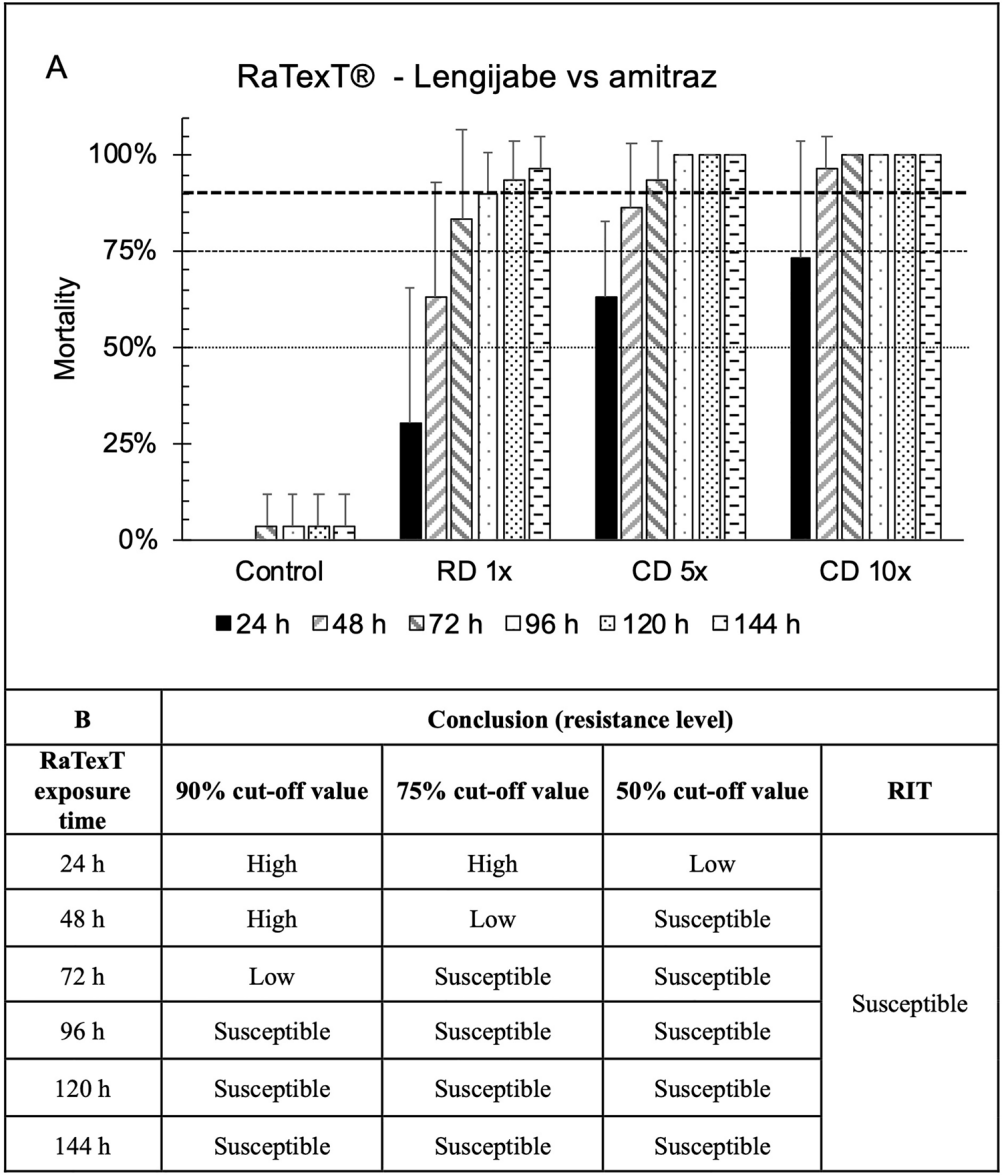
RaTexT^®^ with amitraz, against *R. decoloratus* Lengijabe laboratory strain, at different exposure times and cut-off values for resistance level conclusions (panel **A**. Mortalities; panel **B**. Conclusion)

Furthermore, the comparison between RaTexT^®^ and RIT with amitraz against laboratory tick strains from Tanzania and *R. decoloratus* field ticks from Uganda (panels G and H) is illustrated in Fig. 5. The Lengijabe laboratory strain of *R. decoloratus* was found to be susceptible to amitraz after a prolonged exposure of up to 96 h in RaTexT^®^ (Fig. 5: Panels A and B). However, a similar susceptibility status could not be achieved in the *R. decoloratus* laboratory strain from Monduli within an exposure time of 24 h (Fig. 5: Panels C and D) or in the *R. appendiculatus* laboratory strain from Lushoto within 72 h (Fig. 5: Panels E and F). RaTexT^®^ was able to detect resistance to amitraz in field ticks after an exposure time of 96 h, where the resistance level in RaTexT^®^ was consistently higher than that in RIT (Table 6). The two-proportion Z-Test with *P*-values > 0.01 indicated no significant differences between mortality percentages in 18 out of 72 test comparisons (Table 6). Furthermore, the exposure time for susceptible *R. decoloratus* (Lengijabe) ticks in RaTexT^®^ to amitraz could be shortened when cut-off values for resistance level conclusions were reduced from 90 to 75% (Fig. 6, Panels A and B). Cohen´s Kappa statistical analysis on the entire dataset demonstrated moderate to substantial agreement between RaTexT^®^ and RIT for detecting resistance in cattle ticks when measured at 48 h and 72 h from initial exposure (Table 7).

**Table 6:** Statistical comparison of the percentage mortality of ticks from Tanzania and Uganda in RaTexT^®^ and RIT for amitraz using the two-proportion Z-test combined with the conclusions derived from the decision tree

Tick isolate	Z-test (*P*-value)												Conclusion (resistance level)				
	24 h			48 h			72 h			96 h							
	RD 1×	CD 5×	CD 10×	RD 1×	CD 5×	CD 10×	RD 1×	CD 5×	CD 10×	RD 1×	CD 5×	CD 10×	RaTexT^®^ 24 h	RaTexT^®^ 48 h	RaTexT^®^ 72 h	RaTexT^®^ 96 h	RIT
*R. decoloratus* Lengijabe	< 0.001	0.05*	0.157*	0.06*	0.489*	0.863*	0.445*	0.732*	1*	0.684*	1*	1*	High	Moderate	Low	Susceptible	Susceptible
*R. decoloratus* Monduli	< 0.001	< 0.001	**0.113**	**–**	**–**	**–**	**–**	**–**	**–**	**–**	**–**	**–**	High	**–**	**–**	**–**	Susceptible
*R. appendiculatus* Lushoto	< 0.001	< 0.001	< 0.001	< 0.001	< 0.001	< 0.001	< 0.001	0.003	< 0.001	-	**–**	**–**	High	High	High	**–**	Susceptible
*R. decoloratus* Kyenjojo	**–**	**–**	**–**	< 0.001	< 0.001	0.001	< 0.001	< 0.001	0.001	< 0.001	0.001	0.011	**–**	High	High	High	Low
*R. decoloratus* Wakiso	**–**	**–**	**–**	< 0.001	< 0.001	< 0.001	**–**	**–**	**–**	< 0.001	< 0.001	0.002	**–**	High	**–**	High	Low
*R. decoloratus* Kumi	**–**	**–**	**–**	< 0.001	< 0.001	0.183*	0.002	< 0.001	0.245*	0.002	0.005	0.871*	**–**	High	High	Moderate	Low
*R. decoloratus* Kabarole	< 0.001	< 0.001	< 0.001	< 0.001	< 0.001	< 0.001	< 0.001	< 0.001	< 0.001	< 0.001	< 0.001	0.001	High	High	High	High	Low
*R. decoloratus* Fort Portal	< 0.001	< 0.001	< 0.001	0.001	< 0.001	< 0.001	0.017*	< 0.001	< 0.001	0.109*	< 0.001	0.019*	High	High	High	High	Low

Asterisks indicate *P*-value > 0.01 and no significant differences between tests (RaTexT and RIT)

**Table 7:** Cohen´s Kappa statistical analysis demonstrates the level of agreement between RaTexT® and RIT for detecting resistance in cattle ticks against deltamethrin, cypermethrin/chlorpyriphos/PBO, chlorfenvinphos and amitraz at different exposure times

Acaricide	Exposure time	Number of observations	Observed Kappa	Standard error	0.95 confidence interval	Level of agreement ^*^
Deltamethrin	24 h	8	1	0	1–1	Almost perfect
Cypermethrin/ Chlorpyriphos/ PBO	24 h	4	0.2	0.4	0–0.984	Slight
	48 h	6	0.6667	0.3043	0.0703–1.000	Substantial
	72 h	3	1	0	1–1	Almost perfect
Chlorfenvinphos	24 h	5	0	0	0	None
	48 h	7	0.5882	0.3812	0–1	Moderate
	72 h	3	1	0	1–1	Almost perfect
	96 h	3	1	0	1–1	Almost perfect
Amitraz	24 h	5	0	0.3651	0–0.7157	None
	48 h	7	0	0.5976	0–1	None
	72 h	6	0	0.5774	0–1	None
	96 h	6	1	0	1–1	Almost perfect
Pooled	24 h	22	0.2254	0.1926	0–0.6029	Fair
	48 h	23	0.5106	0.2224	0.0747–0.9465	Moderate
	72 h	14	0.6585	0.2235	0.2204–1	Substantial
	96 h	9	1	0	1–1	Almost perfect

^*^Interpretation of Kappa values according to Cohen (1960), cited by McHugh (2012). Values ≤ 0 indicate no agreement and 0.01–0.20 as none to slight, 0.21–0.40 as fair, 0.41–0.60 as moderate, 0.61–0.80 as substantial, and 0.81–1.00 as almost perfect agreement

The repeatability of RaTexT^®^ with deltamethrin (24 h) was assessed through experiments conducted with the Monduli and Kiboga-2 isolates. Consistently, low mortality was observed across all concentrations, with high relative variability at the lower doses (CV > 70%). Nevertheless, the ICC values were moderately high: 0.665 for Monduli and 0.583 for Kiboga-2, indicating good consistency between independent RaTexT units, despite substantial within-box variability (Table 8).

**Table 8:** Intraclass correlation coefficients (ICC) and descriptive statistics for RaTexT repeatability by concentration, strain, acaricide, and exposure time

Strain	Acaricide	N	Time (h)	Conc	Mean (%)	SD	CV (%)	ICC [95%CI]
Monduli	Deltamethrin	8	24	1×	3.33	3.98	119.52	0.665 [0.574–0.952]
Monduli	Deltamethrin	8	24	5×	3.33	5.35	160.36	
Monduli	Deltamethrin	8	24	10×	5.00	3.98	79.68	
Kiboga-2	Deltamethrin	9	24	1×	10.00	7.26	72.65	0.583 [0.511–0.927]
Kiboga-2	Deltamethrin	9	24	5×	14.44	12.36	85.57	
Kiboga-2	Deltamethrin	9	24	10×	11.48	15.47	134.70	
Lengijabe	Cyper/chlor	6	24	1×	10.56	4.43	41.97	< 10^–6^
Lengijabe	Cyper/chlor	6	24	5×	37.22	20.70	55.61	
Lengijabe	Cyper/chlor	6	24	10×	77.22	17.05	22.08	
Lengijabe	Cyper/chlor	6	48	1×	41.67	20.63	49.51	< 10^–6^
Lengijabe	Cyper/chlor	6	48	5×	92.78	6.47	6.97	
Lengijabe	Cyper/chlor	6	48	10×	98.33	1.83	1.86	
Lengijabe	Cyper/chlor	6	72	1×	72.22	21.36	29.58	< 10^–6^
Lengijabe	Cyper/chlor	6	72	5×	98.89	1.72	1.74	
Lengijabe	Cyper/chlor	6	72	10×	100.00	0.00	0.00	
Kyenjojo	Chlorfenvinphos	7	48	1×	2.32	1.81	77.88	0.011 [0.007–0.117]
Kyenjojo	Chlorfenvinphos	7	48	5×	1.47	1.39	94.18	
Kyenjojo	Chlorfenvinphos	7	48	10×	5.80	2.89	49.79	
Monduli	Chlorfenvinphos	6	24	1×	22.22	8.07	36.33	0.027 [0.017–0.307]
Monduli	Chlorfenvinphos	6	24	5×	49.44	26.11	52.81	
Monduli	Chlorfenvinphos	6	24	10×	73.89	19.25	26.06	
Monduli	Chlorfenvinphos	6	48	1×	56.11	10.42	18.57	< 10^–6^
Monduli	Chlorfenvinphos	6	48	5×	85.00	10.90	12.83	
Monduli	Chlorfenvinphos	6	48	10×	94.44	5.44	5.76	
Kyenjojo	Amitraz	6	48	1×	10.68	5.67	53.09	0.0006 [0.0004–0.0089]
Kyenjojo	Amitraz	6	48	5×	13.56	4.54	33.46	
Kyenjojo	Amitraz	6	48	10×	56.36	10.90	19.34	
Kyenjojo	Amitraz	6	72	1×	10.97	3.17	28.86	0.0003 [0.0002–0.0042]
Kyenjojo	Amitraz	6	72	5×	26.94	9.99	37.11	
Kyenjojo	Amitraz	6	72	10×	78.95	4.38	5.56	

For each combination, the table presents the number of independent replicates (*N*), mean mortality (%), standard deviation (SD), coefficient of variation (CV%), and the intraclass correlation coefficient (ICC) estimated from a linear mixed-effects model (LMM) with its 95% confidence interval (CI). ICC reflects the proportion of variance attributable to differences between independent RaTexT boxes. Only datasets with at least six independent replicates were included in the analysis

The repeatability of cypermethrin/chlorpyrifos/PBO (24–72 h) tests was assessed with the Lengijabe isolate, wherein mortality increased progressively over time, reaching 100% at 10 × after 72 h. However, ICC values remained low across all timepoints (ICC < 10⁻⁶). This suggests minimal detectable variance between boxes, with variation within replicates (Table 8).

For chlorfenvinphos (24–48 h) the repeatability was assessed with the Kyenjojo and Monduli isolates. Low to moderate mortality was observed at 1 × and 5 × , reaching up to 94% at 10 × after 48 h. ICC values ranged from 0.011 to 0.027, indicating low in between-test variability, while CVs ranged from 5 to 95%, depending on the concentration and exposure time (Table 8).

Finally, the repeatability of RaTexT® impregnated with amitraz (48–72 h) was analysed with the Kyenjojo isolate, which showed increasing mortality with more prolonged exposure, with mean values reaching 56.4% at 10 × after 48 h and 78.9% after 72 h. Despite the clear dose–response pattern, ICC values remained very low: 0.0006 at 48 h and 0.0003 at 72 h, with 95% confidence intervals close to zero. These results indicate that nearly all the observed variability occurred within individual RaTexT® boxes, while between-box differences were negligible. Nevertheless, CV values declined at higher concentrations (e.g., 5.6% at 10 × after 72 h), suggesting high internal consistency despite the low ICC (Table 8).

**Fig. 7:**
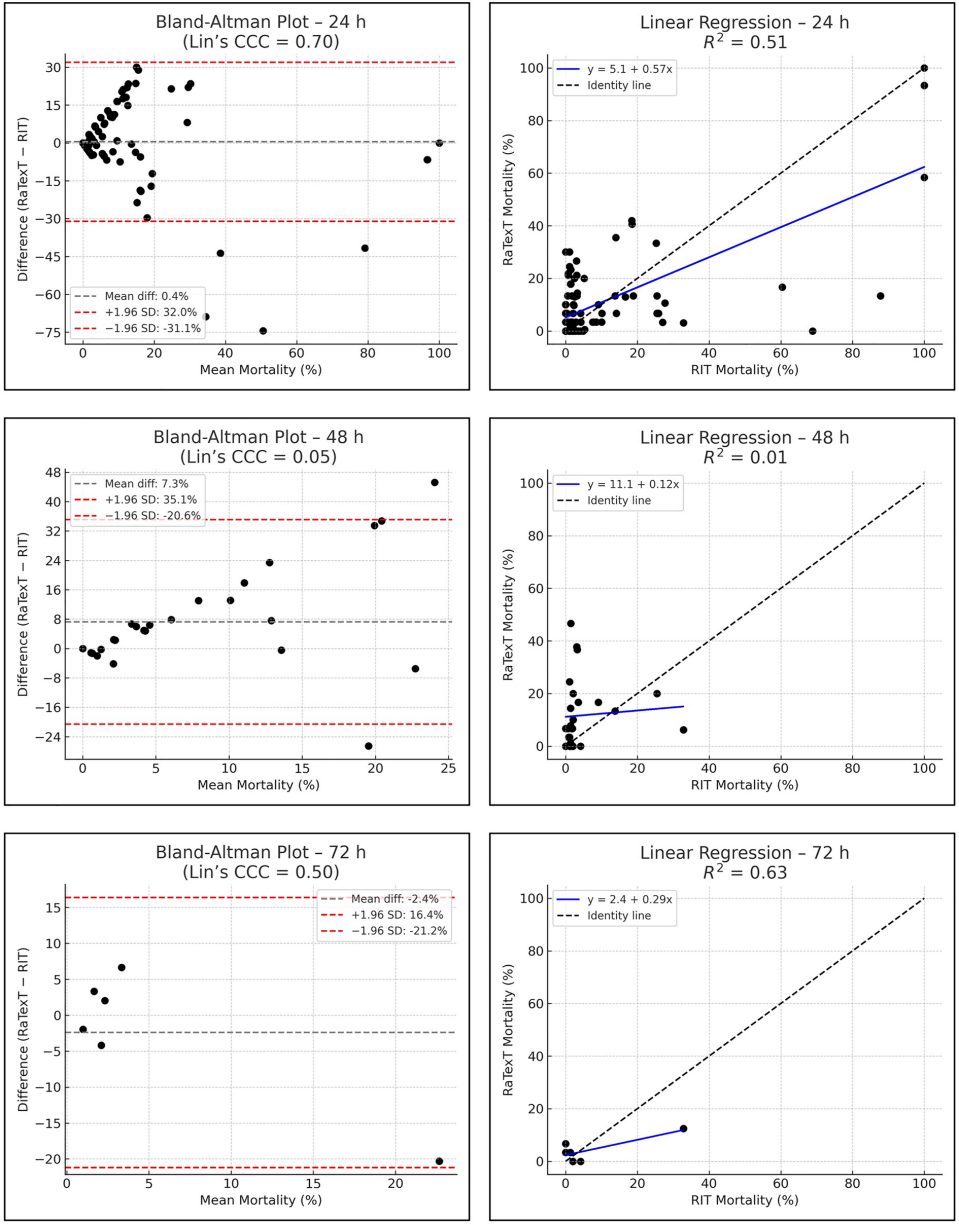
Agreement between RaTexT^®^ and RIT for deltamethrin across different exposure times (24 h, 48 h, and 72 h) by Bland–Altman analysis (left panels) and linear regression (right panels)

**Fig. 8:**
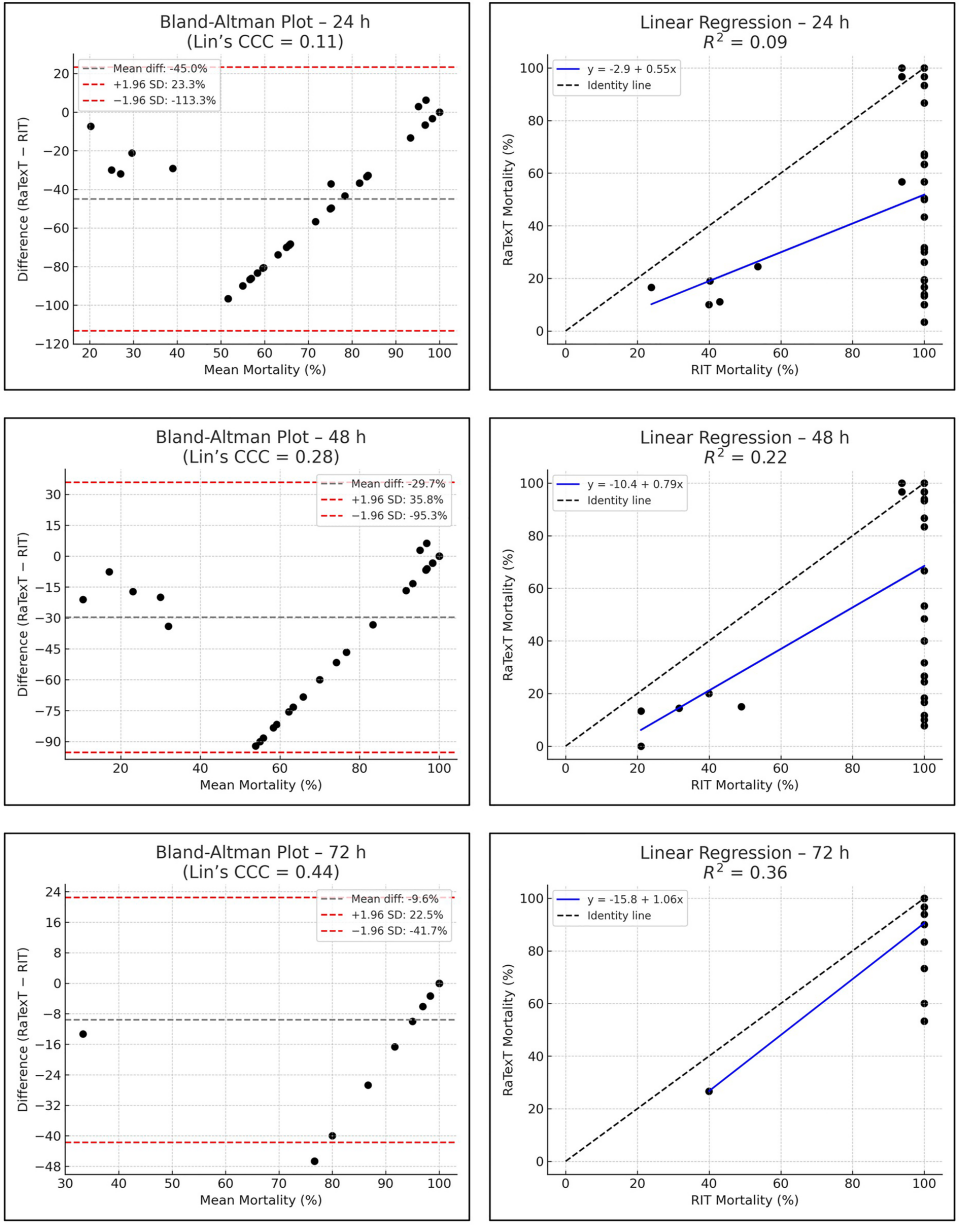
Agreement between RaTexT^®^ and RIT for cypermethrin/chlorpyrifos/PBO across different exposure times (24 h, 48 h, and 72 h) by Bland–Altman analysis (left panels) and linear regression (right panels)

**Fig. 9:**
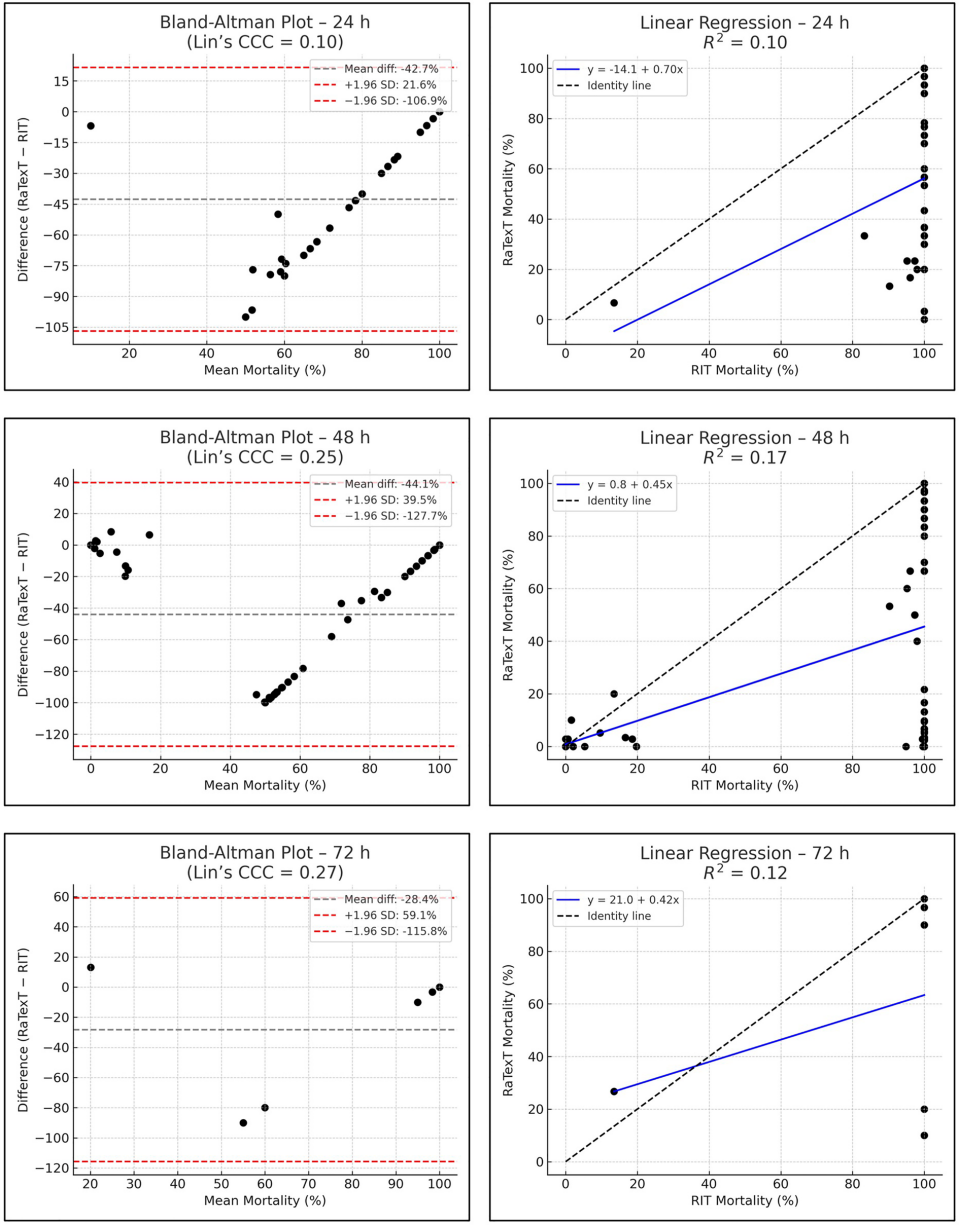

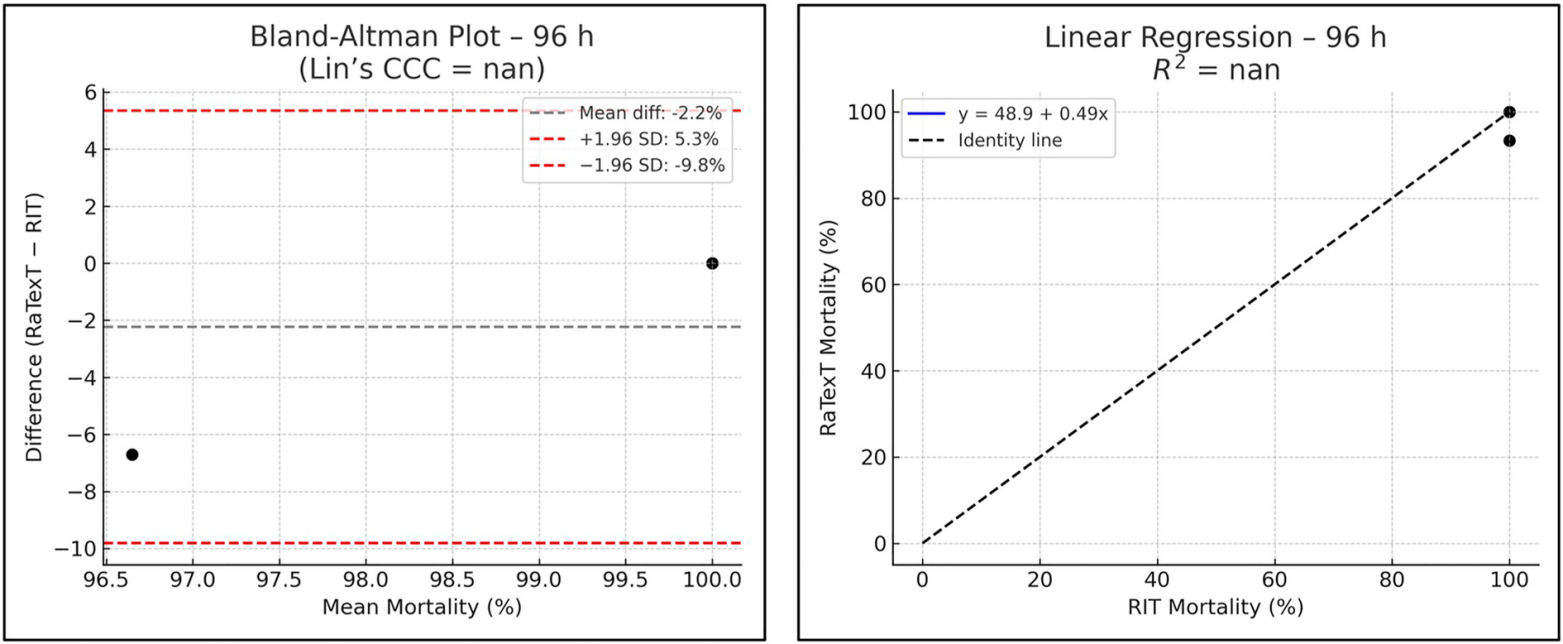
Agreement between RaTexT^®^ and RIT for chlorfenvinphos across multiple exposure times (24 h, 48 h, 72 h, and 96 h) by Bland–Altman analysis (left panels) and linear regression (right panels)

**Fig. 10:**
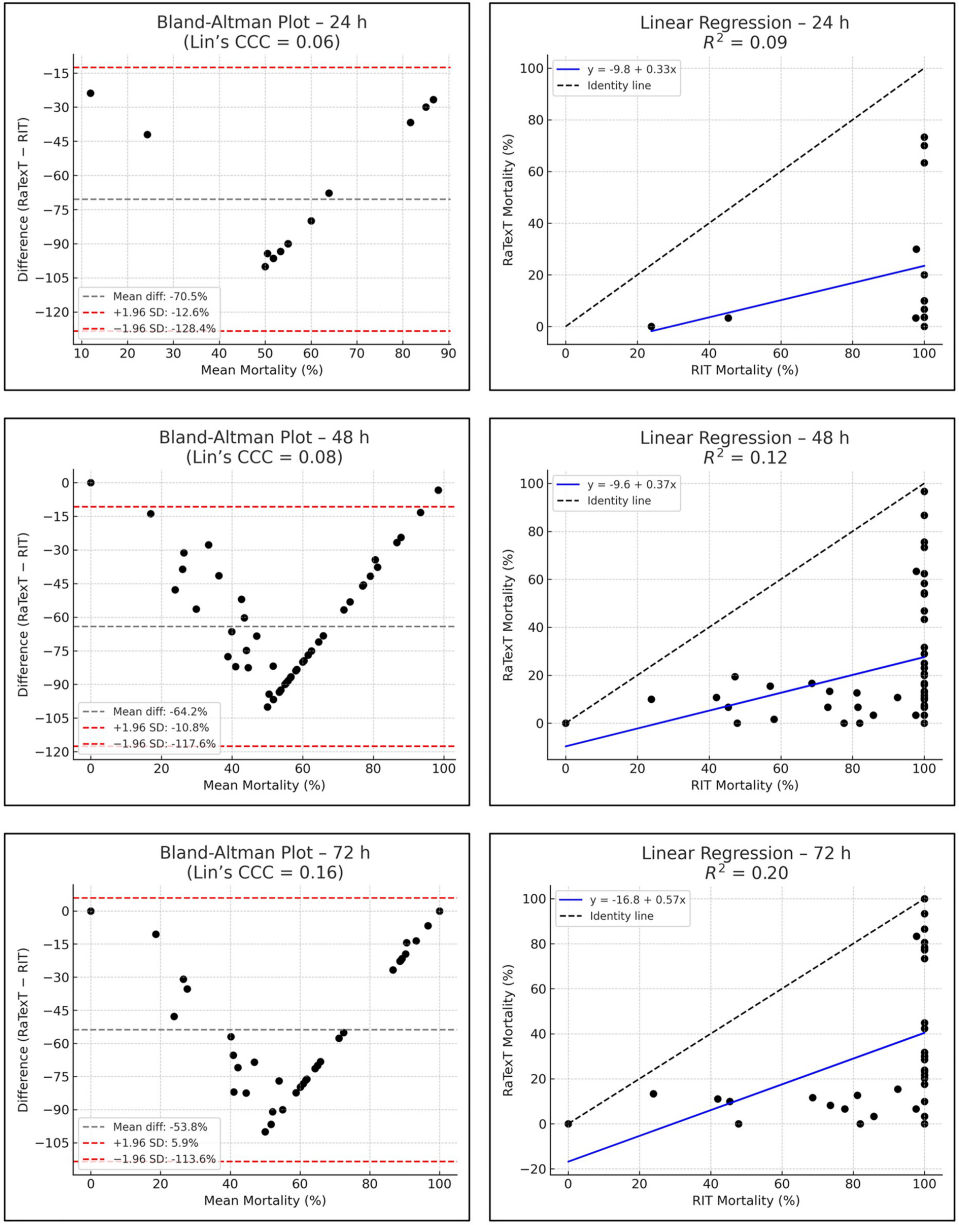

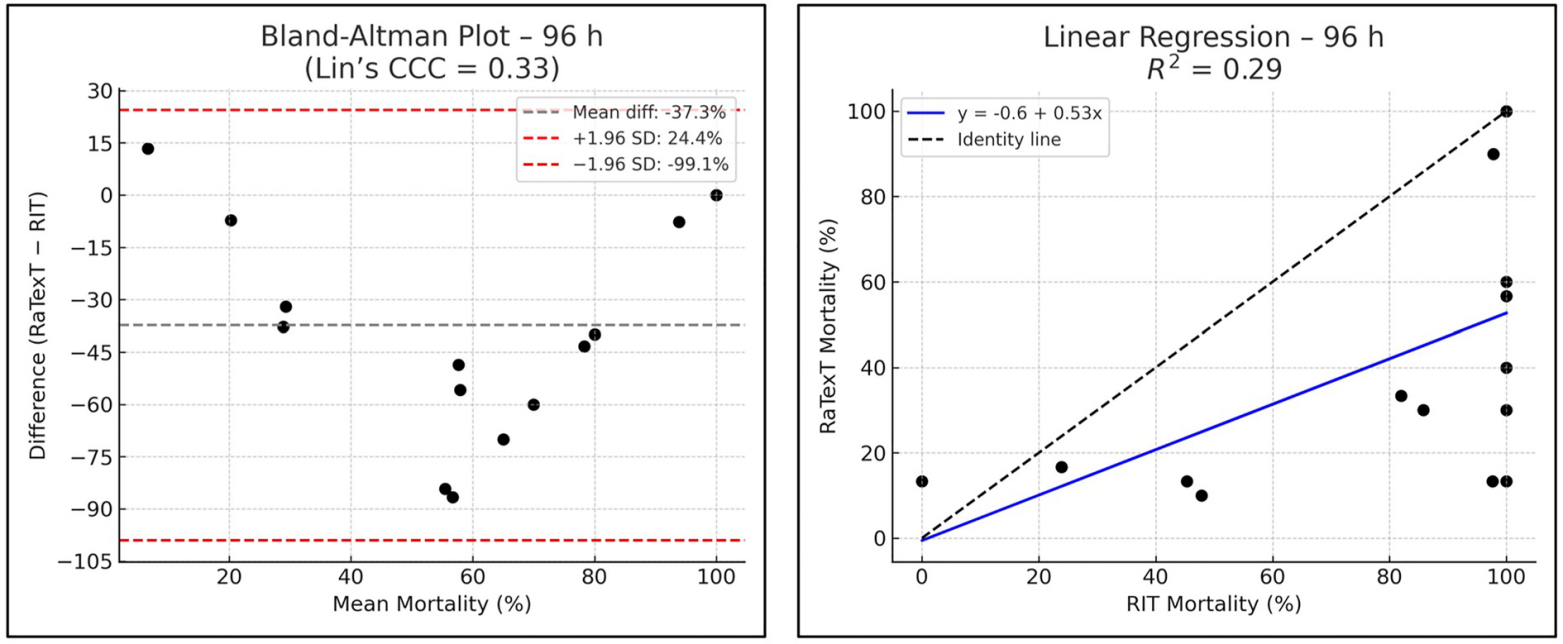
Agreement between RaTexT^®^ and RIT for amitraz across multiple exposure times (24 h to 96 h) by Bland–Altman analysis (left panels) and linear regression (right panel)

The Bland–Altman analysis revealed strong concordance between RaTexT^®^ and RIT for deltamethrin at 24 h (CCC = 0.70; R^2^ = 0.51; bias =  + 0.4%) (Fig. 7). For cypermethrin/chlorpyrifos/PBO the agreement between both tests was low (CCC = 0.11; bias = −45.0%), but improved when observed at 48 h (CCC = 0.28), and yielded the best agreement at 72 h exposure time (CCC = 0.44; R^2^ = 0.36; bias = −9.6%) (Fig. 8). For chlorfenvinphos initial concordance between both tests was low at 24 h and 48 h (CCC = 0.10 and 0.25), but improved towards 72 h (CCC = 0.27) and was highest at 96 h (bias = −2.2%), although CCC and R^2^ could not be computed due to limited number of obervations (Fig. 9). For amitraz, Bland–Altman analysis revealed a poor initial agreement at 24 h (CCC = 0.06; bias = −70.5%). Progressive improvement was observed through 48 h (CCC = 0.08), 72 h (CCC = 0.16), and 96 h (CCC = 0.33; R^2^ = 0.29) (Fig. 10).

## Discussion

The starting point for validating RaTexT^®^ in East Africa was the laboratory validation conducted in Brazil with resistant and susceptible colonies of *R. microplus* ticks [38]. It was found that RaTexT^®^ detected deltamethrin resistance in adult *R. microplus* ticks at the same level as larvae in the RIT [40]. Hence, the same protocol was followed to identify resistance to synthetic pyrethroids in other species of cattle ticks in East Africa. In Tanzania, the existing tick colonies at the Tanzania Plant Health and Pesticides Authority (TPHPA) in Arusha were expanded to accommodate testing of adult and larval ticks from the same colonies. The approach in Uganda was different because tick-rearing facilities were unavailable. Therefore, a hybrid field-laboratory validation was carried out, whereby adult ticks were tested in the field, and the subsequent larvae were tested 6 weeks later at Makerere University in Kampala. The resistance profile of the laboratory colonies in Tanzania had been previously determined using LPT, which confirmed different levels of pyrethroid resistance [23]. It was noticed that pyrethroid-susceptible tick colonies were unavailable at the beginning of the study. However, each batch of RaTexT^®^ was tested before shipment to East Africa to confirm 100% mortality of susceptible ticks exposed to the recommended acaricidal concentration. Furthermore, data obtained with the susceptible Porto Alegre strain were accessible as an outgroup, since the same batch of deltamethrin-impregnated RaTexT^®^ boxes and RIT tests were utilized in Brazil.

Resistance to deltamethrin was detected in Tanzania and Uganda within 24 h by RaTexT^®^ and RIT, with both tests consistently agreeing on the same resistance level determined using the 90% mortality cut-off value. In Tanzania, resistance to deltamethrin was confirmed across consecutive laboratory generations of *R. decoloratus*. Similarly, in Uganda, a high level of deltamethrin resistance detected in RaTexT^®^ in field-collected adult *R. decoloratus* ticks within 24 h was corroborated by finding a similar high resistance level in their larval progeny tested in the RIT. Synthetic pyrethroid resistance is genetically stable due to modifications in the sodium channel, which do not revert to their original configuration, leaving the ticks susceptible again. The frequency of mutations and heritability influence whether ticks within and between farms vary in their resistance profiles. Ultimately, resistance to pyrethroids appears to be independent of the life cycle stage, resulting in matching resistance levels between adult ticks exposed in RaTexT^®^ and larvae in RIT.

Next, it was determined whether RaTexT^®^ can detect resistance to organophosphates, combinations of synthetic pyrethroids and organophosphates, and formamidines (amitraz). It was found that organophosphates require a longer exposure time to affect adult ticks than fast-acting synthetic pyrethroids. When pyrethroid-resistant one-host laboratory ticks were treated with a combination of cypermethrin and chlorpyrifos, extending the exposure time in RaTexT^®^ to 48 h was sufficient, demonstrating their susceptibility to organophosphates. This was confirmed when deltamethrin-resistant *R. decoloratus* ticks used in laboratory experiments in Tanzania were successfully treated with Vectoclor Plus^®^, a blend of cypermethrin and chlorpyrifos (data not shown). In Uganda, field strains of *R. decoloratus* showed high resistance in RaTexT^®^ and moderate resistance in RIT. Combination of compounds like chlorpyriphos and cypermethrin in some acaricide formulations are favored by farmers facing pyrethroid-resistant ticks, which explains their significant market share of 34% in Tanzania and 37.5% in Uganda.

Exposure of adult ticks to chlorfenvinphos in RaTexT^®^ for 48 h revealed that both one-host laboratory tick colonies of *R. decoloratus* in Tanzania were fully susceptible to organophosphates, a finding confirmed by the RIT. Interestingly, the Lushoto strain of *R. appendiculatus,* isolated over 20 years ago in the northwestern part of Tanzania, still exhibited low resistance to chlorfenvinphos in both tests. In Uganda, resistance to chlorfenvinphos was confirmed in field-collected ticks, where the resistance level in RaTexT^®^ consistently exceeded that in the resistance intensity test. Prolonged exposure of field ticks to chlorfenvinphos in RaTexT^®^ did not significantly increase mortality rates, indicating that these ticks were highly resistant. Resistance in *R. appendiculatus* to organophosphates has previously been reported in Uganda [8] and Zimbabwe [51].

Organophosphate toxicity manifests as the inhibition of acetylcholinesterase, resulting in the accumulation of acetylcholine [52]. Organophosphate resistance levels were consistently higher in RaTexT^®^ than in RIT possibly owing to the physiological advantage of fed adult ticks over unfed larvae, which express higher acetylcholinesterase activity. Adult ticks are typically collected after nearly three weeks of feeding. Studies in other arthropods support the hypothesis that pesticide resistance can vary depending on the life stage and feeding status [53]. This emphasizes the importance of utilizing an extended observation period for testing organophosphate-based acaricides in RaTexT® to account for delayed mortality effects. Ultimately, organophosphates continue to play a role, particularly in combination products with synthetic pyrethroids, yet their relative market share remains minimal, estimated at 1.5% in Tanzania and 2.0% in Uganda.

Lastly, RaTexT^®^ was also evaluated for its ability to detect resistance to amitraz. It was found that amitraz acted slowly and required a longer exposure time than chlorfenvinphos. In Uganda, RaTexT^®^ identified amitraz resistance in *R. decoloratus* ticks collected from the field after an exposure time of 96 h, with the resistance level consistently higher than in RIT. In Tanzania, one of the laboratory strains of *R. decoloratus* was found to be susceptible to amitraz after an exposure time of 96 h. However, a similar susceptibility status could not be confirmed in the other laboratory strains because the exposure time in RaTexT^®^ was too short.

Amitraz toxicity is considered to be mediated through the octopamine receptor, although there are several potential related receptors, as well as possible metabolic mechanisms, which lead to rapid detachment and mortality of ticks. Its toxicity to cattle and humans is minimal and less persistent [54]. The detachment of susceptible ticks from treated cattle has made amitraz a popular choice among farmers, as illustrated by its relatively high market shares in Tanzania (23.5%) and Uganda (32.5%). Generally, two types of resistance mechanisms have been proposed: (1) target site insensitivity of adrenergic neuro-receptors (G protein-coupled receptors, beta-adrenergic octopamine receptor, and the octopamine/tyramine receptor) and (2) metabolic detoxification through overexpression of monoamine oxidases and ATP-binding transporters [55–57]. Definitive proof of resistance mechanisms could be obtained by sub-lethal exposure of ticks to amitraz and monitoring the accumulation of mutations across generations. However, a more sophisticated approach could involve genome editing to reverse amitraz resistance to susceptibility in ticks. The uncertainties regarding resistance mechanisms in amitraz complicate the explanation for why larvae in RIT experienced significantly higher mortality rates than adults in the RaTexT^®^. Nevertheless, both tests detected resistance, although the resistance levels differed. This discrepancy could be mitigated by using a less stringent mortality cut-off value of 75%. It can be also postulated that adult ticks are a more suitable target for resistance detection and that resistance in larvae tends to be underestimated.

The overall statistical comparison between the two tests using the two-proportion Z-test (P > 0.01) indicated that there were no significant differences in percentage mortality across 72 out of 168 comparisons (42.9%). This is quite favorable considering that different numbers of two tick stages were compared. Notably, Cohen´s Kappa statistical analysis of the entire dataset demonstrated moderate to substantial agreement between RaTexT^®^ and RIT in detecting resistance in cattle ticks after 48 h and 72 h of tick exposure. Cohen’s kappa interrater reliability remains to be established to demonstrate that the results are independent of the individual interpreting the test [65].

In terms of RaTexT^®^ repeatability, it was observed that most ICC values were low, especially for cypermethrin/chlorpyrifos and chlorfenvinphos, which reflect limited variability, particularly when mortality outcomes were highly homogeneous (e.g., 100% across replicates). These results highlight that low between-box variance actually signals high assay stability.

The comparative agreement analyses presented in this study reveal that the diagnostic performance of RaTexT^®^ is closely linked to the mode of action profile of each acaricide class. For fast-acting compounds such as deltamethrin, high concordance with the RIT was observed as early as 24 h, supporting the suitability of RaTexT^®^ as a rapid resistance screening tool. In contrast, compounds with slower onset of action, such as chlorfenvinphos and amitraz, would require extended exposure times to achieve alignment between methods. The moderate improvement seen for the cypermethrin/chlorpyrifos/PBO combination suggests that synergistic or mixed-mode formulations may require intermediate incubation durations to reveal their full effect in adult ticks. These findings reinforce the need for acaricide-specific RaTexT^®^ protocols, tailored to the expected time of action and physiological target. Notably, the lower concordance observed at early time points for organophosphates and formamidines possibly reflects the higher physiological tolerance of engorged adult ticks compared with larvae.

Currently, no molecular tests are available as alternatives to bioassays for rapidly determining acaricide resistance in livestock ticks. A recent encouraging attempt was made to develop a PCR-based assay, which is not yet commercially available [58]. Interestingly, although many molecular markers have been described, [59] most are unsuitable for monitoring acaricide resistance except for Kdr, a well-established genetic mutation in synthetic pyrethroids [60, 61].

RaTexT^®^ could be used in studies where a correlation between bioassays and in vivo efficacy trials needs to be established [62–64]. RaTexT^®^ can also be tailored to the range of products in a specific local market. These factors are considered in the final design of RaTexT^®^, which is anticipated to encompass seven distinct acaricidal classes or combinations, including macrocyclic lactones. Given that the latter are frequently overused for tick control, this has led to undesirable side effects of resistance in nematodes and ticks[66].

## Conclusions

RaTexT^®^ is the most rapid, pen-side test available for managing acaricide resistance in livestock ticks. The correlation between RaTexT^®^ and RIT was most consistent for synthetic pyrethroids,. Levels of organophosphate resistance were consistently higher in RaTexT^®^ than in RIT, maybe owing to the physiological advantage of fed adult ticks, which express higher acetylcholinesterase activity than unfed larvae. For amitraz, RaTexT^®^ identified resistance in adult ticks only after prolonged exposure, which could be shortened with a mortality criterion less stringent than 90%. Cohen´s Kappa statistical analysis of the entire dataset demonstrated moderate to substantial agreement between RaTexT^®^ and RIT in detecting resistance in cattle ticks between 48 and 72 h of tick exposure. Together, continuous agreement analyses (Bland–Altman, CCC, regression) and categorical measures (Z-test, Kappa) support the utility of RaTexT^®^ as a proxy for RIT, provided that test conditions are optimized for each acaricide class.

## Supplementary Information

Additional file 1.

## Data Availability

Data supporting the main conclusions of this study are included in the manuscript.

## Abbreviations

RaTexT^®^: Rapid Tick exposure Test
RIT: Resistance intensity test
LPT: Larval packet test
PBO: Piperonyl butoxide
