# Supplementary material for: Diagnostic performance of a Rapid Tick exposure Test (RaTexT®) to detect acaricide resistance in cattle ticks in East Africa
Source: Parasit Vectors. 2025 Aug 11;18:342. doi: 10.1186/s13071-025-06995-6 (PMC12337526; doi:10.1186/s13071-025-06995-6)
Supplement: Supplementary file 1 — Additional file 1. [file 13071_2025_6995_MOESM1_ESM.docx]

**SUPPLEMENTARY DATA**

Table 1. Summary of agreement analyses between RaTexT® and RIT for deltamethrin across different exposure times. This table presents the results of Bland-Altman analysis, Lin’s Concordance Correlation Coefficient (CCC), and linear regression comparing RaTexT® and Resistance Intensity Test (RIT) mortality data for deltamethrin. The mean difference (bias), limits of agreement (LoA), CCC values, regression coefficients, and R² are shown for each exposure time point (24 h, 48 h, and 72 h).

| Time (h) | Mean Difference (%) | Lower LoA (%) | Upper LoA (%) | Lin’s CCC | R² | Regression Slope | Regression Intercept |
| --- | --- | --- | --- | --- | --- | --- | --- |
| 24 | 0.43 | -31.08 | 31.95 | 0.70 | 0.51 | 0.57 | 5.13 |
| 48 | 7.26 | -20.61 | 35.12 | 0.05 | 0.01 | 0.12 | 11.15 |
| 72 | -2.41 | -21.19 | 16.37 | 0.50 | 0.63 | 0.29 | 2.35 |

# **Table 2. Summary of agreement analyses between RaTexT® and RIT for cypermethrin/chlorpyrifos/PBO.**

| Time (h) | Mean Difference (%) | Lower LoA (%) | Upper LoA (%) | Lin’s CCC | R² | Regression Slope | Regression Intercept |
| --- | --- | --- | --- | --- | --- | --- | --- |
| 24 | -44.99 | -113.30 | 23.31 | 0.11 | 0.09 | 0.55 | -2.93 |
| 48 | -29.72 | -95.25 | 35.82 | 0.28 | 0.22 | 0.79 | -10.42 |
| 72 | -9.61 | -41.69 | 22.48 | 0.44 | 0.36 | 1.06 | -15.81 |

# This table presents the results of Bland-Altman analysis, Lin’s Concordance Correlation Coefficient (CCC), and linear regression comparing RaTexT® and RIT mortality data for the cypermethrin/chlorpyrifos/PBO combination. Values are shown for each exposure time point (24 h, 48 h, 72 h).

# **Table 3. Summary of agreement analyses between RaTexT® and RIT for chlorfenvinphos.**

| Time (h) | Mean Difference (%) | Lower LoA (%) | Upper LoA (%) | Lin’s CCC | R² | Regression Slope | Regression Intercept |
| --- | --- | --- | --- | --- | --- | --- | --- |
| 24 | -42.68 | -106.93 | 21.57 | 0.10 | 0.10 | 0.70 | -14.10 |
| 48 | -44.08 | -127.71 | 39.55 | 0.25 | 0.17 | 0.45 | 0.78 |
| 72 | -28.35 | -115.84 | 59.14 | 0.27 | 0.12 | 0.42 | 20.98 |
| 96 | -2.23 | -9.82 | 5.35 |  |  | 0.49 | 48.88 |

# This table summarizes the Bland-Altman bias, 95% limits of agreement (LoA), Lin’s Concordance Correlation Coefficient (CCC), and linear regression results comparing RaTexT® and RIT for chlorfenvinphos at each exposure time. Missing CCC and R² values at 96 h reflect insufficient sample size for correlation analysis.

**Table 4. Summary of agreement analyses between RaTexT® and RIT for amitraz.**

| Time (h) | Mean Difference (%) | Lower LoA (%) | Upper LoA (%) | Lin’s CCC | R² | Regression Slope | Regression Intercept |
| --- | --- | --- | --- | --- | --- | --- | --- |
| 24 | -70.50 | -128.43 | -12.56 | 0.06 | 0.09 | 0.33 | -9.75 |
| 48 | -64.21 | -117.61 | -10.82 | 0.08 | 0.12 | 0.37 | -9.65 |
| 72 | -53.85 | -113.58 | 5.89 | 0.16 | 0.20 | 0.57 | -16.82 |
| 96 | -37.35 | -99.09 | 24.40 | 0.33 | 0.29 | 0.53 | -0.56 |

This table presents the results of Bland-Altman analysis, Lin’s CCC, and linear regression comparing RaTexT® and RIT for amitraz. Values reflect increasing agreement over time, particularly at 96 h, in line with the delayed mode of action of formamidines.
